# *Caenorhabditis* nematodes influence microbiome and metabolome characteristics of their natural apple substrates over time

**DOI:** 10.1128/msystems.01533-24

**Published:** 2025-01-10

**Authors:** J. Johnke, J. Zimmermann, T. Stegemann, D. Langel, A. Franke, L. Thingholm, H. Schulenburg

**Affiliations:** 1 Zoological Institute, Kiel University, Kiel, Germany; 2 Max Planck Institute for Evolutionary Biology, Ploen, Germany; 3 Botanical Institute, Kiel University, Kiel, Germany; 4 Institute of Clinical Molecular Biology, Kiel University, Kiel, Germany; Swansea University, Swansea, United Kingdom

**Keywords:** microbiome assembly, microbiome functions, *Caenorhabditis elegans*, apple metabolome

## Abstract

The microbiomes of host organisms and their direct source environments are closely linked and key for shaping microbial community dynamics. The relationship between these linked dynamics is largely unexplored because source substrates are usually unavailable. To address this current knowledge gap, we employed bacteriovorous *Caenorhabditis* nematodes as a unique model system, for which source substrates like rotting apples can be easily collected. We compared single host microbiomes with their corresponding apple source substrates, as well as nematode-free substrates, over a 2-year sampling period in the botanical garden in Kiel, Germany. We found that single worms have unique microbiomes, which overlap most strongly with nematodes from the same source apple. A comparison to previous, related work revealed that variation in microbiome composition of natural *Caenorhabditis* isolates is significantly influenced by the substrate type, from which worms were obtained (e.g., fruits or compost). Our current sampling further showed that microbiome assembly is mostly driven by dispersal limitation. Importantly, two independent analysis approaches consistently suggest that worm microbiomes significantly influence characteristics of the apple microbiomes, possibly indicating niche construction by nematodes. Moreover, combining apple microbiome and metabolome data, we identified individual microbes and specific compounds indicative of fruit ripening that are significantly associated with nematode presence. In conclusion, our study elucidates the complex relationship between host microbiomes and their directly connected substrate microbiomes. Our analyses underscore the significant influence of nematode microbiomes on shaping the apple microbiome and, consequently, the fruit’s metabolic capacity, thereby enhancing our general understanding of host–microbiome interactions in their natural habitat.

**IMPORTANCE:**

Almost all complex organisms are host to a microbial community, the microbiome. This microbiome can influence diverse host functions, such as food processing, protection against parasites, or development. The relationship between host and microbiome critically depends on the assembly of the microbial community, which may be shaped by microbes in the directly linked environment, the source microbiome. This assembly process is often not well understood because of the unavailability of source substrates. Here, we used *Caenorhabditis* nematodes as a model system that facilitates a direct comparison of host and source microbiomes. Based on a 2-year sampling period, we identified (i) a clear link between assembly dynamics of host and source microbiomes, (ii) a significant influence of nematode microbiomes on apple microbiomes, and (iii) specific microbes and compounds that are associated with the presence of nematodes in the sampled substrates. Overall, our study enhances our understanding of microbiome assembly dynamics and resulting functions.

## INTRODUCTION

The intricate relationship between hosts and their microbiomes is well-established, with host fitness being significantly influenced by the composition and functioning of the associated microbial communities (1–4). Animals feeding on fiber-rich diets rely on their microbial symbionts to process food, such as ruminants (5, 6) or termites (7). The microbiome is critical for the development of diverse animals, including, for example, the cnidarian *Hydra vulgaris* (8), the bobtail squid (9), and also affects the maturation of the mammalian immune system (10). The microbiome can also provide protection against infecting pathogens, as described for human hosts (11), or the nematode *Caenorhabditis elegans* (1, 12). Interestingly, the composition of microbiomes often varies substantially among individual hosts of the same species (13–16). This variation can result from differences in host diet (17, 18), physiology (19, 20), genetics (21–23), and also random processes during assembly of the microbiome, such as the initial frequencies relevant for priority effects and environmental availability of microbes (24–27). Such within-host variation is likely to influence the expressed functions of the particular microbiome (28–30). Therefore, it is of particular importance to understand which factors shape within-host variation in microbiome composition and characteristics.

Four high-level processes can explain microbial assembly: (i) movement of organisms across space (dispersal), (ii) deterministic fitness differences between individuals (selection), (iii) stochastic changes in relative abundances (drift), and (iv) generation of genetic variation (diversification) (31, 32). For microbiomes, the assembly process is additionally influenced by hosts with their specific life history traits, like lifespan, growth, motility, or food processing, which themselves can further shape the environment in which the microbiome assembly takes place. Hosts’ influence and control of microbiomes vary across host systems, and similarities in microbiome composition of related hosts can be explained by host filtering *or* shared evolutionary history (33, 34). The directly connected environmental microbiome (i.e., the substrate microbiome) is of interest because it can serve as the source microbiome from which microbes colonize a host. Therefore, a comparison between host-associated microbiomes and substrate microbiomes is likely critical to assess the particular influence of host and environment on the microbiome assembly process, the involved community dynamics, and its inter-individuality.

The current study aims to decipher the ecological underpinnings of host–microbiome associations by characterizing the relationship between host and corresponding substrate microbiomes. We used *Caenorhabditis* nematodes as an informative model. These nematodes acquire food and additional gut-colonizing bacteria from rotten plant material, like apples. Both the nematodes and the inhabited apples can be easily collected, enabling a comparison of single host microbiomes with the microbial community of their directly connected substrates. These comparisons were used to obtain insights into the variation in microbiome composition dynamics between these two sample types (i.e., worms and substrates) and to assess the directionality of the influence of nematode and substrate microbiomes upon one another. Additionally, we also characterized host-free apples, allowing us to assess which bacteria, abiotic characteristics, and also metabolites govern the presence of *Caenorhabditis* nematodes in their natural environment.

## RESULTS

### Overview of study design, main research questions, and compared data

**Fig 1:**
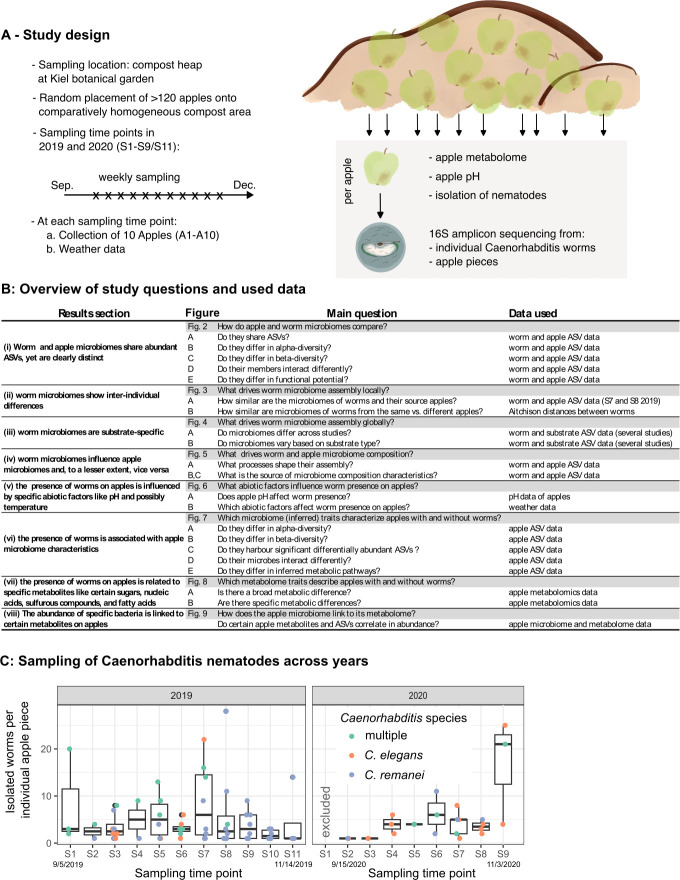
Overview of study design and questions. (A) Study design and sample processing. Apples were evenly spread across a compost heap. Every week, 10 apples (A1–A10) were randomly removed from the compost heap and analyzed in the laboratory. Pieces of these apples were used for apple metabolomics, pH measurement, worm isolation, and 16S amplicon-based microbiome characterizations. For *Caenorhabditis* worm isolation, apple pieces were placed into petri dishes and covered with viscous medium, leading worms to float to the top of the medium. Worms were collected with a pipette, washed in a washing buffer, and added separately to wells of a 96-well plate for DNA isolation. Worm species identity was determined via diagnostic PCRs for *Caenorhabditis* species. (B) Overview of study questions and used data. The table further indicates the results sections and the figures that display the results. A complete overview, containing (statistical) methods and references to other figures and tables can be found in Table S1. (C) Number of worms found per apple for all sampling time points in 2019 (from 5 September 2019 to 14 November 2019) and 2020 (from 15 September 2020 to 3 November 2020). Each circle represents an apple sample on which *Caenorhabditis* nematodes were found. *Caenorhabditis* species are indicated by the color of the circles, with green circles representing apples on which multiple *Caenorhabditis* species were present.

Apples, and subsequently worms, were sampled weekly from a compost heap in the Kiel Botanical Garden during the autumns of 2019 and 2020 (i.e., September until December; Fig. 1A), resulting in 11 sampling dates in 2019 and nine in 2020, followed by characterization of the microbiomes of individual worms and apple substrates. We additionally collected basic information on abiotic factors during sampling and characterized the metabolome of the sampled apples (Fig. 1A). Based on this material and the obtained data, we addressed fundamental questions on worm microbiome assembly and characteristics of worm-harboring apples (Fig. 1B; more details in Table S1). More specifically, the available material for our analyses included 16S amplicon sequencing data for the microbiomes of 170 and 87 individual *Caenorhabditis* nematodes, along with that of 82 and 83 apple substrates from 2019 and 2020, respectively (Table S2). Fifty-seven of the sequenced apples contained *Caenorhabditis* nematodes of various species, including *C. elegans* (18), *C. remanei* (26), both *C. briggsae* and *C. remanei* (1), *C. elegans* and *C. remanei* (10)*,* and all three *Caenorhabditis* species (2) (all known from previous work to coexist in northern Germany [35]). A total of 108 apple samples did not contain any *Caenorhabditis* worms or had incomplete information on worm presence and were excluded from analyses requiring such information. The nematodes were most abundant in 2019, with worm-positive apple samples yielding up to 28 individually characterized worms (Fig. 1C). We then compared microbiome data from worms and apples to (i) identify differences and similarities and to characterize the factors determining (ii) local and (iii) global differences between microbiomes, and (iv) assess the processes shaping the assembly of worm and apple microbiomes (e.g., comparison of worm microbiomes with their corresponding source apple microbiomes; Fig. 1B; Table S1). Since not all apples harbored worms, we assessed whether the presence of *Caenorhabditis* nematodes is associated with (v) specific abiotic factors, (vi) apple microbiome characteristics, and (vii) metabolome traits (Fig. 1B; Table S1). Finally, we explored (viii) the association between apple microbiome abundance data and apple metabolite abundance data (Fig. 1B; Table S1). In the following sections, we present the results of our analyses.

#### Worm and apple microbiomes share abundant ASVs, yet are clearly distinct

**Fig 2:**
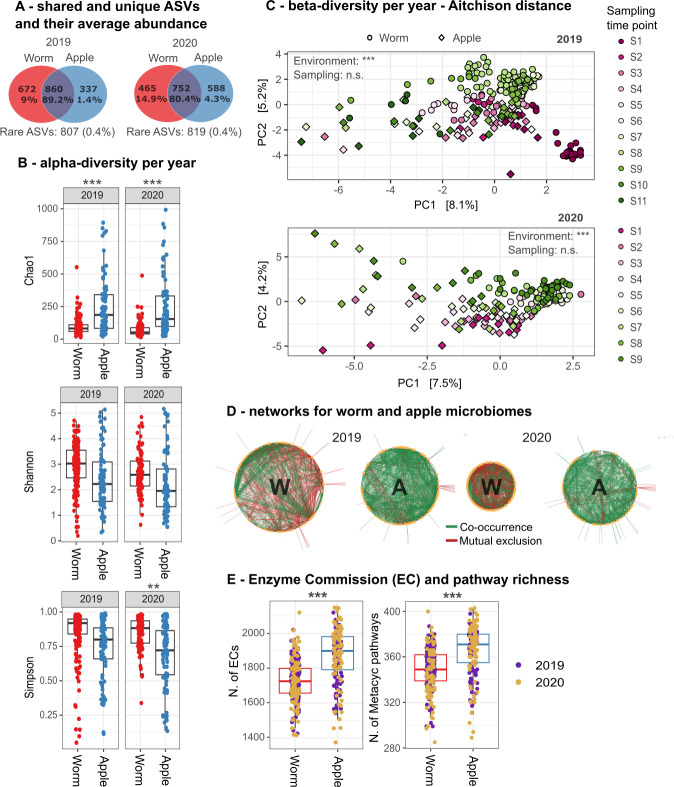
*Caenorhabditis* and apple microbiomes show significant differences across 2019 and 2020. (A) Number of shared and unique ASVs between apple and *Caenorhabditis* worm microbiomes and their average abundance across samples in percent. Rare ASVs are below 0.1% relative abundance. (B) Alpha-diversity metrics for apple and worm microbiomes (i.e., environment). Each dot represents a sample. A linear mixed-effects model was used to assess the relationship between alpha-diversity and environment, with apple number or sampling time point as a random factor to account for dependencies. Asterisks indicate the Benjamini–Hochberg (BH)-adjusted *P*-value for the “environment” term (see Table S4 for details). (C) Beta-diversity shown as Aitchison distance between any two samples. Colors denote sampling time points, with circles for worm samples and diamonds for apple samples. Statistical analysis was performed using the adonis2 function (Aitchison distance ~environment or sampling time point), with particular apples as random factor (strata) to account for dependencies of worms that were isolated from the same apple. See Table S6 for more details. (D) CoNet-generated networks from apple and worm microbiomes. Only significant associations are shown. Green lines indicate co-occurrence, and red lines indicate mutual exclusion of two ASVs. Network size scales with the number of nodes and edges. Node size scales with the number of edges per node. (E) Enzyme commission (EC) and MetaCyc pathway richness inferred by PICRUSt2. Each dot represents a sample. A linear mixed-effects model was used to assess diversity differences between environments, with apple per sampling time point as a random factor to account for dependencies. Asterisks indicate BH-adjusted *P*-values for the “environment” term. See Table S15 for further statistical details.

We used the 16S amplicon data of single worms and apple pieces to assess similarities and variation among these sample types. We found that worm and apple microbiomes share the majority of abundant bacterial taxa, always inferred as amplicon sequencing variants (ASVs) (Fig. 2A; Table S3). At the same time, many of the less abundant ASVs are specific for worm or apple samples. This observation indicates that worm microbiomes are directly connected to their source microbiome, while also representing distinct environments, enabling different bacteria to thrive and coexist and/or be selected by the nematode. It should be noted that apple and worm samples differed in their biomass, which can introduce a bias towards higher diversity and, hence, more unique ASVs in the larger apple samples. However, worms are moving freely on apples and can thereby gather microbes from a larger radius. Worm microbiome assembly processes are thus most likely strongly influenced by the microbes found in a larger sample, as the apple pieces characterized here. Importantly, our analysis did not reveal more unique ASVs in apple microbiomes, indicating that the larger biomass did not *per se* cause a larger ASV diversity.

Our next objectives were to use alpha-diversity measures in order to assess variation between worms and substrates in ASV richness (indicated by Chao1) or ASV richness and evenness (indicated by Shannon and Simpson diversity). We further analyzed beta-diversity in order to assess general differences in microbiome composition between sample types, using Aitchison distances (providing a measure of Euclidian distance between two multi-dimensional compositions) and Unifrac distances (a distance metric that considers the relative relatedness of community members). The alpha-diversity analysis revealed that apple microbiomes are significantly more species-rich (high Chao1), but less even (low Shannon and Simpson, Fig. 2B; Fig. S1A top panel, Table S4). Richness and evenness were found to increase over sampling time, and this increase was negatively correlated with maximum temperature of the environment (Fig. S2; Table S1). Beta-diversity analysis confirmed the significant differences in the community composition per environment (apple vs worm), identified using both Aitchison (Fig. 2C; Table S6) and UniFrac distances (Fig. S3; Table S7). These apple–worm differences are based on specific community members belonging mainly to the *Enterobacteriaceae* and *Acetobacteriaceae* (top panel Fig. S1A and B). In the analysis of beta-diversities, worm samples also cluster by *Caenorhabditis* species (Fig. S4A; Table S8), but overall clustering strongly suggests that the observed variation between the microbiomes of different *Caenorhabditis* species is due to the variation caused by sampling time points and sampling years (Fig. S4B; Table S8). Taken together, our analysis highlights that despite the overlap of abundant ASVs between worm and apple microbiomes, both are clearly distinct and vary over time.

In line with these findings, we identified (i) indicator ASVs for apple and worm microbiomes (based on specificity, i.e., how exclusively an ASV is associated with apples or worms, and fidelity, i.e., how frequently the ASV occurs within apples or worms; Table S9), (ii) significant differentially abundant ASVs across these groups of sample types, calculated using DeSeq2 (Fig. S5), and (iii) core microbiomes, defined as ASVs that appear in at least 80% of the samples per sample type (Table S10). Overall, apple microbiomes are characterized by more indicator ASVs (1,719 ASVs), significantly differentially abundant ASVs (compared with worms), and share a greater core (nine ASVs across years and two ASVs for 1 year, respectively) than worm microbiomes (indicator ASVs: 160, core: one ASV across years, one for 2019). Most core ASVs belong to the *Acetobacteraceae* and are related to the previously defined synthetic microbiome community for *C. elegans*, CeMbio, consisting of either 12 or 43 (extended CeMbio) bacteria of the globally most abundant *C. elegans* microbiome members (36, 37).

We next extended our analysis to co-occurrence networks as an indicator for bacterial interactions in order to explore variation between worm and substrate microbiomes. We found that the nodes of the networks (which can be a single ASV or a higher taxonomic level) often consist of core and/or indicator ASVs and that highly connected nodes often belong to the *Acetobacteraceae* (Fig. 2D; Table S11). Further, worm microbiomes show far more negative edges (i.e., connections) than apple microbiomes (2019: 37% vs 11% and 2020: 47% vs 8% neg. edges, Table S12), indicating more negative associations between worm ASVs or higher taxonomic levels, possibly suggesting increased competition. An ecological explanation could be that the limited resources within the worm gut, compared with the source apple, resulted in the mutual exclusion of ASVs with overlapping niche breadths, which is consistent with the lower ASV richness that we observed in worms (Fig. 2B).

Since phylogenetically similar bacteria are expected to have more overlapping functional potentials (38) and, therefore, more overlapping niche breadths, we compared the Faith phylogenetic diversity (PD) between apple and worm microbiomes and found significantly higher PD in apple microbiomes. However, apple microbiomes are more diverse than worm microbiomes, and this might explain the observed differences in PD. We therefore repeated this analysis, limiting it to apple and worm samples with exactly matching Chao1 values (i.e., matching richness; Fig. S6; Table S13) and found no significant differences in PD. This indicates that ASV richness correlates with PD. However, only few samples have matching Chao1 values (11 in 2019 and 20 in 2020). To extend our analysis, we tested if apple or worm microbiomes, respectively, have a higher or lower PD than expected by chance by computing Chao1-based null distributions from worm and apple samples (see Materials and Methods for details). We found that worm samples are significantly less phylogenetically diverse than expected by chance (2019: *P* = 0.0154, 2020: *P* = 0.0126; two-sided *P*-values after BH correction; Table S14). The opposite is true for apple samples (2019: *P* < 0.0001, 2020: *P* = 0.002; two-sided *P*-values after BH correction; Table S14). This indicates that factors like environmental filtering and a restricted resource range lead to a phylogenetically more similar worm microbiome and that higher habitat complexity and niche competition are likely to account for the higher PD in apples.

In line with this, it has been proposed that higher community biomass (as it should be the case for apple microbiomes) should result in an increased importance of niche differences of the members of a community (39). In contrast, when bacteria face high filtering upon initial worm gut colonization, niche differences, and hence PD, become less important due to a decrease in density and community richness. Indeed, we found that functional potential (used here as a proxy for niche differences), as inferred from our ASV data by PICRUSt2 using Enzyme Commission (EC) enzymes and MetaCyc pathways, is smaller in worm communities than in apple communities (Fig. 2E; Table S15). To account for the potential correlation between alpha diversity and functional potential, we computed random null distributions for EC and MetaCyc pathway richness for worm and apple samples based on Chao1 range (as before). We found that apple samples have significantly higher EC and MetaCyc pathway richness than expected by chance (2019: EC *P* = 0.0000, Metacyc *P* = 0.0004, 2020: EC *P* = 0.0056, MetaCyc *P* = 0.0136; two-sided *P*-values after BH correction; Table S15). In contrast, worm microbiomes have a lower EC and MetaCyc pathway richness, which, however, was only significantly lower than expected by chance in case of EC richness in 2019 (2019: EC *P* = 0.0036, MetaCyc *P* = 0.0980, 2020: EC *P* = 0.1318, MetaCyc *P* = 0.2138; two-sided *P*-values after BH correction, Table S15). This indicates that the higher ASV richness of apple microbiomes translates directly to a higher functional potential, whereas a similar relationship could not be inferred for worm microbiomes.

When looking closer at the different pathway subsystems of the PICRUSt2 results, we found biosynthesis, degradation, and energy–metabolism pathways to be enriched in apple microbiomes (Fig. S7; Table S16). This indicates a higher complexity and resource richness of the apple environment. According to a recent publication, more complex resource conditions favor multiple steady community states via stochastic assembly processes and the hierarchical structure of consumer–resource interactions (40). Indeed, we observed differences in apple communities, even when apples were sampled at the same time. These differences in source communities should translate to the worm microbiome, assuming that all microbes are derived from a common source pool. This is a possible explanation for the observed individuality of worm microbiomes (see below).

#### Worm microbiomes show inter-individual differences

**Fig 3:**
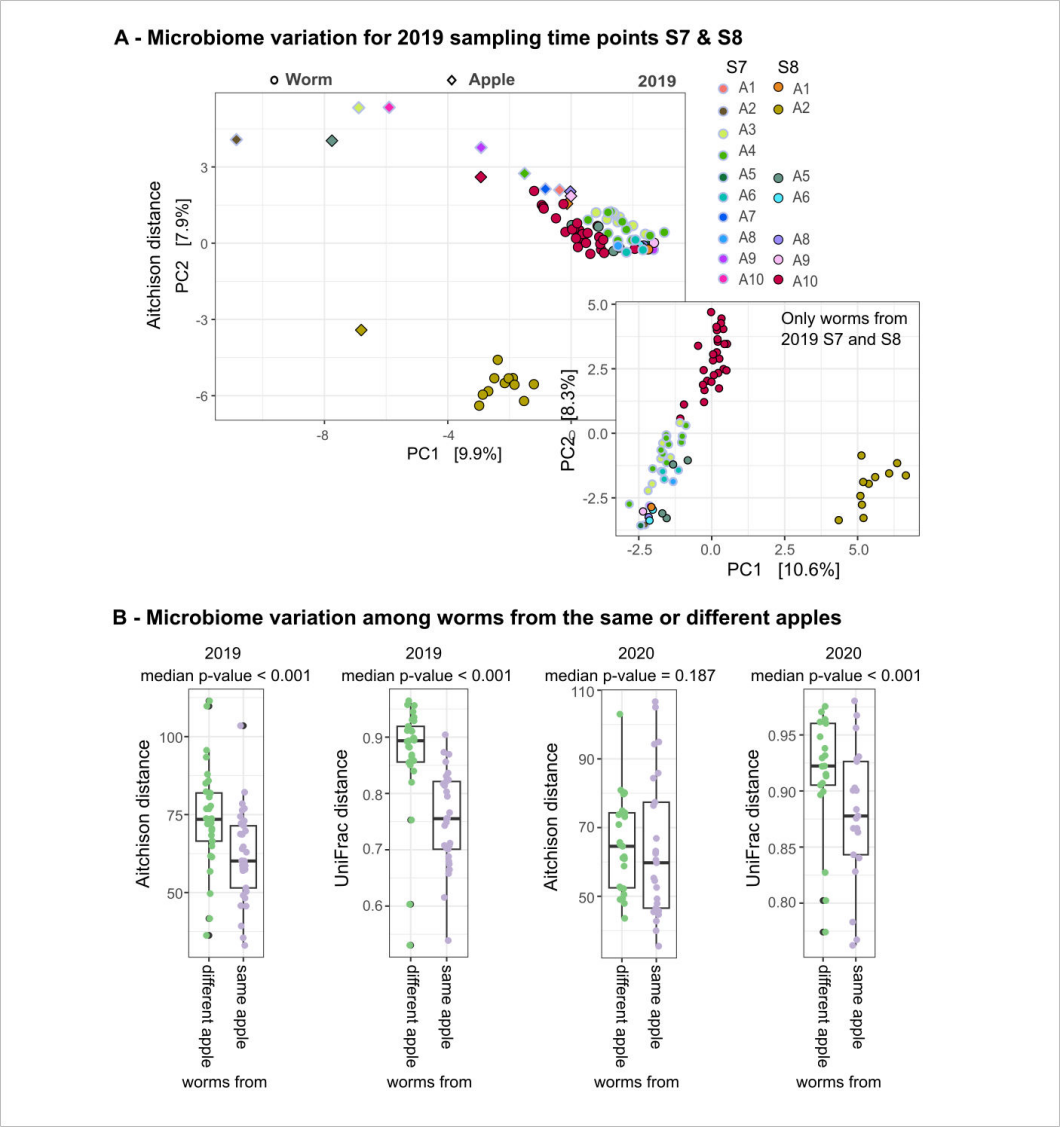
High inter-individuality of *Caenorhabditis* microbiomes in 2019 and 2020. (A) Beta-diversity shown as Aitchison distance for two sampling time points (S7 and S8) in 2019. *Caenorhabditis* worm samples (circles) and apple samples (diamonds) are colored by their respective source apple (A1–A10). Dark contours indicate samples from S8. Identical numbers across sampling time points do not represent the same apples. The inset highlights beta-diversity among worm samples, showing close clustering of worm microbiomes from the same and different apples. (B) Distances between paired worm microbiomes, calculated using Aitchison or UniFrac distance, for worms from the same or different apples in 2019 and 2020. For each focal worm, we selected a single random comparison either with a worm from the same apple or a worm from a different apple, ensuring that each worm was included only once in the analysis. To capture the variability of all possible pairings, we repeated this random selection process 100 times and calculated a *P*-value for each iteration using a paired Wilcoxon test. Each figure panel presents one possible outcome with the median *P*-value from these repeated tests on top of each panel. The distribution of all *P*-values is shown as a histogram in Table S17.

Consistent with our above findings, worm microbiomes differed substantially in composition, even when sampled from the same apple, which we here illustrate with the results for two sampling time points (S7 and S8 from 2019; Fig. 3A) and an overall analysis (Fig. 3B). Still, worms sampled from different apples also show substantial overlap in their microbiomes (inset Fig. 3A). Together, these observations indicate a common framework of microbiome assembly (and overlaps in the substrate community) but with different possible outcomes. Yet, despite the inter-individual differences, worm microbiomes from the same apple are overall more similar than those from different apples (Fig. 3B; Table S17).

Our next aim was to assess how individuality of worm and apple microbiomes is influenced by ongoing decay of the apple substrate across time. We thus assessed variation of worm and apple microbiomes over the time that the apples have spent on the compost heap within each year, using Aitchison distances between or within the different environments (i.e., worms and apple substrates). We found an increase in Aitchison distance over time on compost within each year (Fig. S8; Table S18). This result indicates that apples that stayed longer on the compost heap had more diverged microbiomes than those from early time points and also harbored worms with more distinct microbiomes.

#### Worm microbiomes are substrate-specific

**Fig 4:**
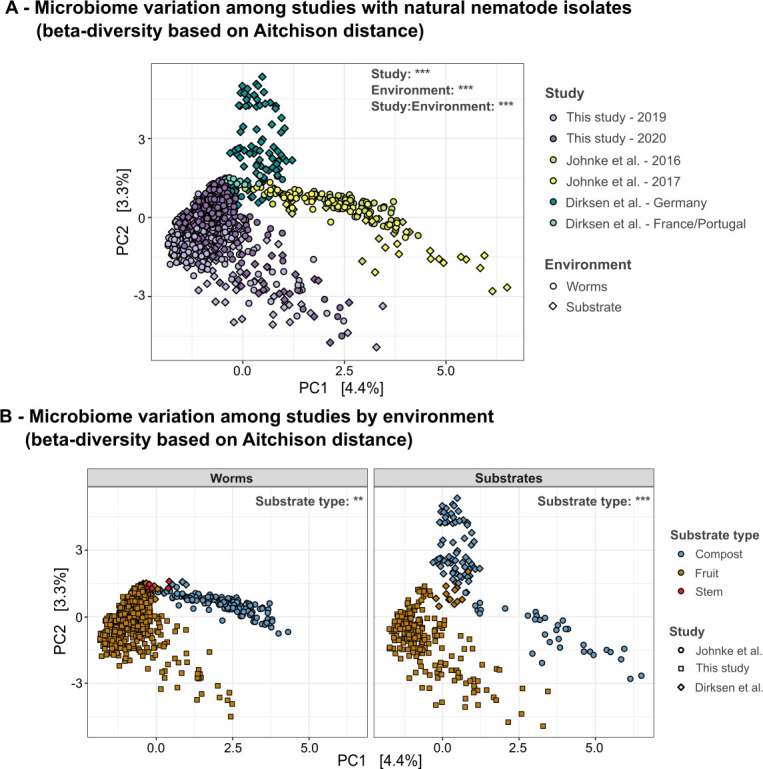
Microbiome characteristics are associated with sampled substrate types across independent studies. (A) Beta-diversity (Aitchison distance) across studies on the *Caenorhabditis* microbiome. Environment can be either worms (circles) or associated substrate samples (e.g., compost, fruit, plant stem; shown as diamonds), and both are colored by study. The Dirksen et al. study was split by country to account for geographical differences. The other two studies collected all materials in the Kiel Botanical Garden. Statistics were performed using the adonis2 function (Aitchison distance ~study * environment). See Tables S19 and S20 for statistical and posthoc results. (B) Beta-diversity across studies by environment (worm vs substrate), with shapes indicating different studies and colors showing substrate type. Worm and substrate results are in separate panels. Statistics were performed using adonis2 (distance ~substrate type, strata = study) to account for non-independent samples. Details are in Table S19.

As a next step in our analysis, we took advantage of the only two previous data sets on the microbiome of natural isolates of *Caenorhabditis* nematodes and associated substrates, with the aim to assess to what extent microbiome characteristics are affected by year, different substrate samples, and also study. The two previous studies were performed by the laboratory of Marie-Anne Félix (part of the study by [21]) and us (both studies). The studies used similar substrates as our current study (i.e., fruits) but also other substrate types (i.e., compost material). The general methods for processing material and molecular analysis were highly similar, especially for the current study and that by (13), where the main work was performed by the same person. This comparison now revealed significant community differences by study and environment (i.e., worm or apple substrate; Fig. 4A; Fig. S1A; Tables S19 and S20). We identified distinct indicator ASVs for the different studies, but also observed shared indicator ASVs across studies (Table S21). Interestingly, 20 indicator ASVs, most from the *Acetobacteraceae*, are shared between the current study and that by Dirksen et al., which considered fruit and compost substrates from the same location in Germany and other locations in Europe (21). Further, worm and substrate microbiomes can be distinguished based on substrate type (i.e., compost, fruit, and stem), from which nematodes and associated substrate material were isolated (Fig. 4B; Table S19). Specifically, apple samples were mostly characterized by the presence of bacteria belonging to the *Acetobacteriaceae* (Fig. S1A), and bacteria from this family cluster separately in the taxonomic ordination plot (Fig. S1C). We further identified different indicator ASVs for the distinct substrate types and few indicator ASVs for combined substrate types (Table S22). For instance, only for worm samples, we observed two indicator ASVs for fruit and compost samples, both belonging to the *Enterobacterales*. Further, we found one core ASV (i.e., an ASV that is present in at least 80% of the samples) for the fruit substrate samples (ASV 669, *Tatumella*), but not for compost samples or worm samples from specific substrates.

Overall, our analysis shows that worm microbiomes are substrate-specific, suggesting that the composition of the worm microbiome is linked to the substrate environment that harbors the nematodes.

#### Worm microbiomes influence apple microbiomes and, to a lesser extent, vice versa

**Fig 5:**
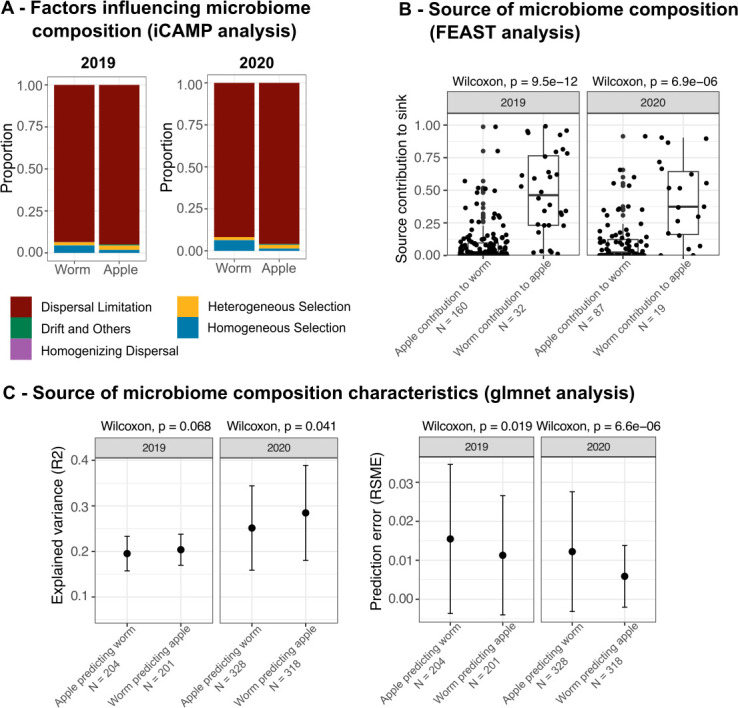
The *Caenorhabditis* microbiome more strongly influences the apple microbiome, while both are mainly shaped by stochastic processes. (A) iCAMP results showing contribution of different processes to microbiome assembly in *Caenorhabditis* nematodes and apples. Stochastic processes include dispersal limitation (red), drift and others (green), and homogenizing dispersal (purple), while deterministic processes are heterogeneous (yellow) and homogeneous selection (blue). Exact proportions are in Table S24, with detailed bin statistics in Table S25 (2019) and Table S26 (2020). (B) FEAST-based predictions of source-sink dynamics of worm microbiomes, and their source apple microbiome indicates a greater contribution of the worm microbiome to the apple microbiome than vice versa. The significance of a difference between the two alternatives was tested using the Wilcoxon signed-rank test with *W*_2019_ = 503 and *W*_2020_ = 280. (C) glmnet-based predictions of source-sink dynamics show root mean square error (RMSE) and *R*² (explained variance), both supporting a stronger influence of the worm microbiome on the apple microbiome in both years. Statistical differences between the respective alternatives were assessed using the Wilcoxon signed-rank test with *W*_2019_ = 17,728 and *W*_2020_ = 41,467.

Our next aim was to understand the factors that drive microbiome assembly in worms and apples over time. Given the observed similarities between worm and apple microbiomes and their divergence over time on compost, we hypothesized that the worm microbiomes are closely linked to their direct substrate microbiome and that the linked microbiomes influence each other. As a first step, we assessed the relative importance of stochastic and deterministic (i.e., random vs non-random) effects on microbiome assembly, using two null model approaches implemented in R packages iCAMP and NST, which are both able to employ phylogenetic information of microbiome community members. While NST calculates the overall proportion of stochastic and deterministic processes, iCAMP further sub-categorizes stochastic processes into “Dispersal limitation” (i.e., restricted dispersal), “Drift and others” (i.e., random fluctuations in species abundance due to chance events), and “Homogenizing dispersal” (i.e., dispersal that leads to a reduction of community difference), while deterministic processes are divided into “Heterogeneous selection” (leading to variability in community composition) and “Homogeneous selection” (leading to less variability in community composition). We found that worm and apple microbiomes were predicted to be strongly assembled via stochastic processes, with worm microbiomes significantly more than apple microbiomes (Fig. S9; Table S23). Dispersal limitation was the most dominant stochastic process across phylogenetic bins (Fig. 5A; Table S24 to S26). The most dominant deterministic process was homogeneous selection.

Since worm samples cluster rather with their respective source substrate than with worms from other substrate types when comparing different studies (see above), we next wondered to what extent apple microbiomes influence characteristics of worm microbiomes and vice versa. For this analysis, we took advantage of our study design, where all samples were collected from the same compost heap and should thus have been shaped by the same general environmental conditions, and where we could compare the worm microbiomes with that of their directly connected apple substrate (from which worms were isolated). We addressed this question by applying two methods. First, we used the source tracker FEAST to estimate the contribution of source communities to a sink community by an expectation–maximization approach (41). By comparing the explanatory power of apple samples as sources for worm samples as sinks and vice versa, we found that the contribution of worm microbiomes to the composition of their directly linked apple microbiomes was higher than the contribution of apple microbiomes to worm microbiomes (Fig. 4B). Second, we employed a penalized regression model to predict worm ASV abundances by apple abundances and vice versa and compared their predictive power. For this, we randomly assigned pairs consisting of a single worm microbiome sample and its true source apple. This process was repeated 100 times to obtain an ASV table of paired worm and apple samples with the same dimensionality. This was necessary since we isolated multiple worms from single apples. Next, we predicted the abundances of each ASV in apples (or worms) by all ASV abundances from worms (or apples). Finally, we took the mean of the predictive performance (RSME, R2) for each ASV across the 100 random assignments. Again, the observed variance in apple microbiomes was found to be better explained by worm ASVs than vice versa, and with a lower root mean square error (RSME) (Fig. 4C).

Overall, these results strongly suggest that worm microbiomes at least partially shape the composition of the substrate apple microbiomes and thereby directly influence their habitat and food source.

#### The presence of worms on apples is influenced by specific abiotic factors like pH and possibly temperature

**Fig 6:**
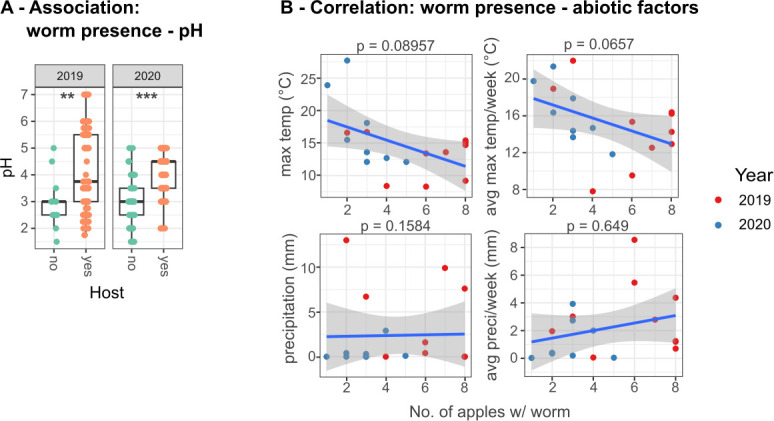
Variation in pH and maximum temperature is associated with the presence of *Caenorhabditis* nematodes. (A) pH levels of sampled apples with or without worms in 2019 and 2020. A linear mixed-effects model analyzed differences in log-transformed pH data across different apples, using the sampling time point as a random variable to account for dependencies. Asterisks indicate a significant difference between samples with or without *Caenorhabditis* nematodes. Details are provided in Table S27. (B) The relationship between the number of apples with worms (*x* axis) and maximum temperature, average maximum temperature per week, precipitation, and average precipitation per week (all *y* axes) during the sampling periods in 2019 (red dots) and 2020 (blue dots). The blue lines indicate the best fit lines for a linear relationship between both variables, while the shaded areas indicate the 95% confidence intervals for the predicted values. A linear mixed-effects model assessed statistical differences in the number of apples with worms based on the respective abiotic factors, with year as a random factor to account for dependencies. Detailed statistics are presented in Table S28.

Worm and apple microbiome assembly processes appear to be closely linked. We therefore asked whether worm-free and worm-harboring apples have distinct characteristics, and which environmental factors determine worm presence. We used available weather data for the respective sampling time points and apple pH measurements and correlated them with worm presence. Our analysis revealed that nematode presence is significantly associated with high apple pH and shows a statistical trend (i.e., 0.1 < *P* < .05) for low maximum temperature of the environment (Fig. 6A and B; Table S27 and 28). The pH range of apples, on which worms were found, increased over the time that the apples spent on the compost heap (Fig. S10A; Table S29). In contrast, variation in average and minimum temperature, precipitation, and barometric pressure of the environment was not significantly associated with nematode presence (Fig. S10B; Table S28). Together, these observations suggest that pH and environmental temperature could affect the likelihood of worm presence on an apple.

#### The presence of worms is associated with apple microbiome characteristics

**Fig 7:**
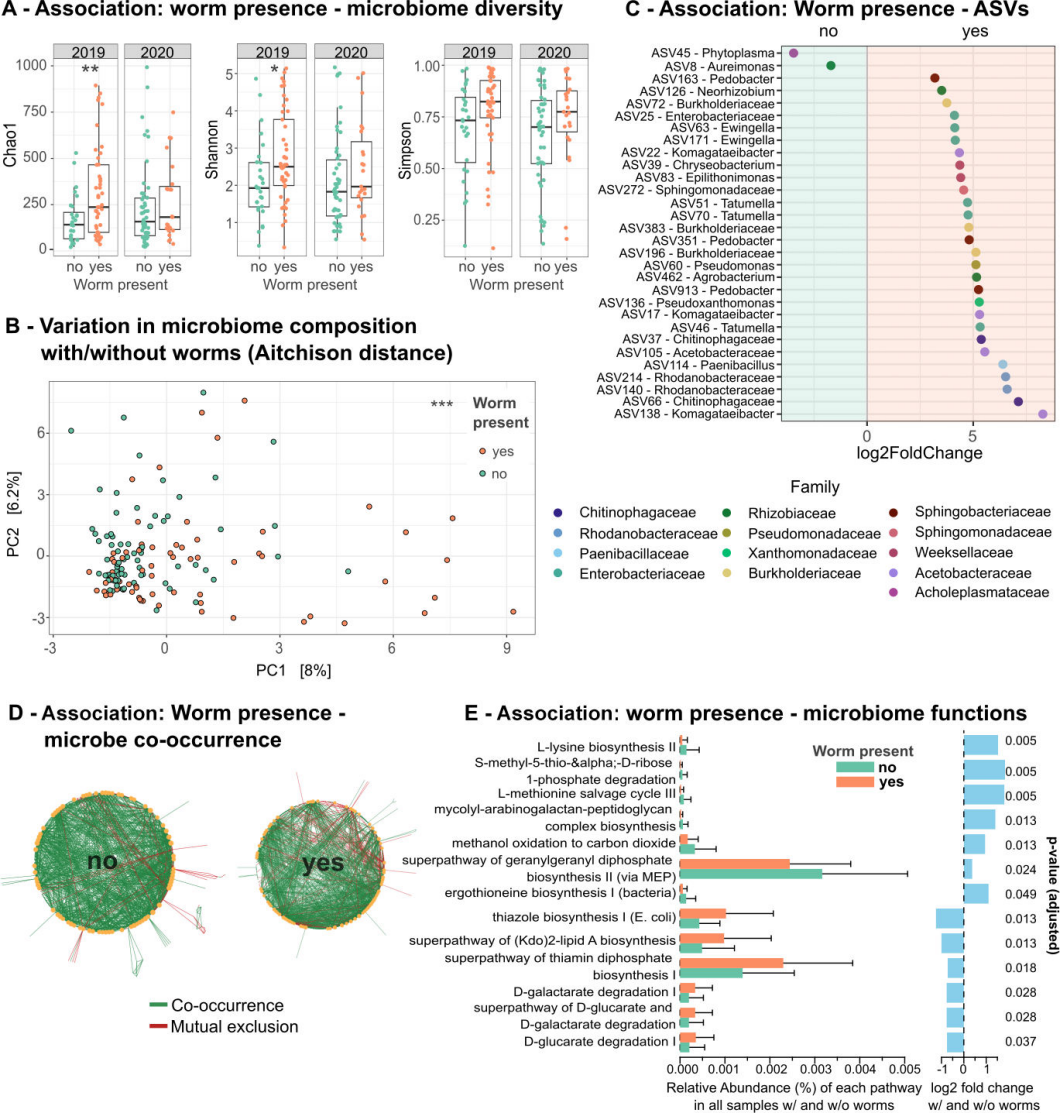
The presence of *Caenorhabditis* nematodes is significantly linked with specific bacterial ASVs and associated metabolic capacities. (A) Variation in alpha diversity between apples with and without worms in 2019 and 2020. The different panels show different alpha diversity measures (always on Y axis), including Chao1, Shannon, and Simpson diversity. A linear mixed-effects model assessed statistical differences, accounting for sampling time as a random factor. Asterisks indicate significant differences between worm-harboring and worm-free apples within each year (detailed statistics in Table S30). (B) Beta diversity, represented as Aitchison distance between samples, distinguishes apples with and without *Caenorhabditis* worms, analyzed using the adonis2 function from the vegan package, with sampling time as a random factor (details in Table S31). (C) Differentially abundant ASVs identified via Deseq2 show ASVs more abundant in worm-free apples (“no”) versus those more abundant in worm-containing apples (“yes”), indicated by different colors for taxonomic families. (D) Co-occurrence networks as inferred with CoNet of microbiome communities from apples with or without worms (yes and no, respectively). Only significant associations are shown. Green lines indicate co-occurrence, red lines indicate mutual exclusion of two ASVs. Details are given in Table S36. Network size scales with the number of nodes and edges. Node size scales with the number of edges per node. (E) Significant differentially abundant MetaCyc pathways between apple microbiomes with (orange bars) and without worms (green bars) based on PICRUSt2 predictions, with log2 fold change indicated by blue bars. ALDEx2 and a Wilcoxon rank test were used for statistical analysis, with BH-adjusted *P*-values shown in blue on the right (details in Table S37).

We next wondered whether a specific composition of the apple microbiome predicts the presence or absence of worms and identified a significant effect of alpha- and beta-diversity (Fig. 7A and B; Tables S30 and S31). In detail, worm-harboring apples have a more diverse (in terms of richness, i.e., Chao1, and richness and evenness, i.e., Shannon and Simpson diversity) and distinct (Fig. 7C) microbiome. Two ASVs are more abundant on worm-free apples and 28 ASVs on apples harboring worms. We did not find a significant association between single ASVs and nematode presence using a linear mixed-effects model and accounting for pH changes and the specific apples per sampling time point as random factor when considering the sampling years separately, but found three significant associations with ASVs when analyzing the years jointly (Tables S32 to S34). These ASVs belong to the *Acetobacteraceae* and *Burkholderiaceae*. In addition, we did not find a significant difference in the distance between the worm microbiomes and either the worm-free or the worm-harboring apple microbiomes (Fig. S11A and B).

Co-occurrence networks generated from worm-harboring apple microbiomes have proportionately more negative edges (Fig. 7D; Table S36, 14.8% vs 7.7%), possibly indicating a negative correlation between taxa and thereby competition. However, the number of PICRUSt2 inferred KEGG enzymes (ECs) and MetaCyc pathways is not different between apple samples with and without worms (data not shown) indicating similar functional potentials of the two types of apple microbiomes. We still find that certain pathways are significantly different in their abundance in apple microbiomes with and without worms (Fig. 7E; Table S37). Specifically, the microbiomes of worm-free apples are enriched for processes like biosynthesis of L-lysine, mycolyl-arabinogalactan-peptidoglycan complex, geranylgeranyl diphosphate, and ergothioneine, the degradation of S-methyl-5-thio-alpha-D-ribose 1-phosphate, the L-methionine salvage cycle III, and the methanol oxidation to carbon dioxide, whereas the microbiome of worm-containing apples are associated with the biosynthesis of thiazol, (Kdo)2-lipid A, thiamin diphosphate, and D-galacterate and D-glucarate degredation.

Taken together, these results suggest that worm presence either significantly impacts apple microbiomes or that worms only thrive on apples with a specific microbiome.

#### The presence of worms on apples is related to specific metabolites like certain sugars, nucleic acids, sulfurous compounds, and fatty acids

**Fig 8:**
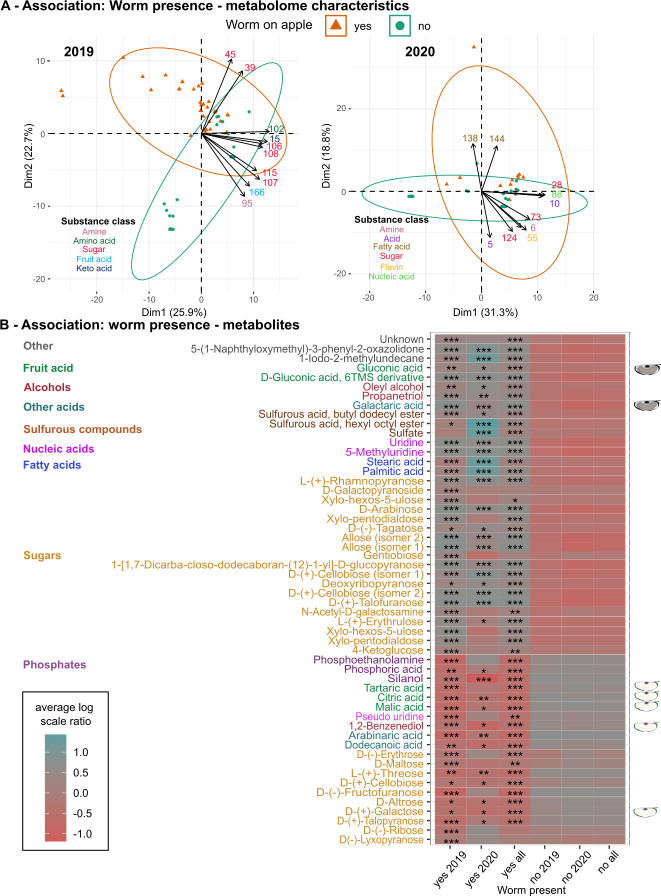
Specific metabolites and substance classes are significantly associated with apples harboring *Caenorhabditis* worms. (A) Principal component analysis of the metabolome of apples from 2019 and 2020. Each shape refers to a separate apple sample. Apples with *Caenorhabditis* worms are shown as orange triangles and those without worms as green circles. Ellipses of the same color indicate the positioning of the samples within the two dimensions shown, while arrows highlight the top 10 metabolites influencing this positioning and colors indicating their substance class. Metabolite identities and their values for the first five components are provided in Table S38. (B) Average metabolite abundances (scaled and normalized; metabolites shown along vertical axis) for apples with and without worms and for both years separately and combined (across horizontal axis). Asterisks indicate whether a metabolite’s abundance significantly depends on the presence or absence of worms and are only given in the “yes” columns of the heatmap. Statistical significance was determined with a linear mixed-effects model, with all *P*-values BH adjusted. Further details are given in Table S39. Only highly significant metabolites (***) from any analysis are shown. Font colors indicate substance classes, and apple pictograms denote metabolites previously associated with fresh or ripe fruit.

To further elucidate the relationship between worm presence and apple characteristics, we extended our genome-based functional predictions by directly measuring and comparing the metabolomes of worm-free and worm-associated apples. This comparison revealed that apple metabolomes cluster depending on worm presence in a PCA (Fig. 8A; Table S38). This clustering is mainly driven by sugars (2019) and fatty acids (2020). We further determined metabolites that differ significantly in abundance in worm-free and worm-associated apples in a year or in both years using a linear mixed-effects model (Fig. 8B; Table S39). Again, we found distinct sugar levels in these sample types, and most of the significant metabolites show higher abundance in worm-associated apples. These findings suggest that worms are usually associated with apples that contain higher amounts of certain sugars, nucleic acids, sulfurous compounds, and fatty acids, but less phosphates.

#### The abundance of specific bacteria is linked to certain metabolites on apples

**Fig 9:**
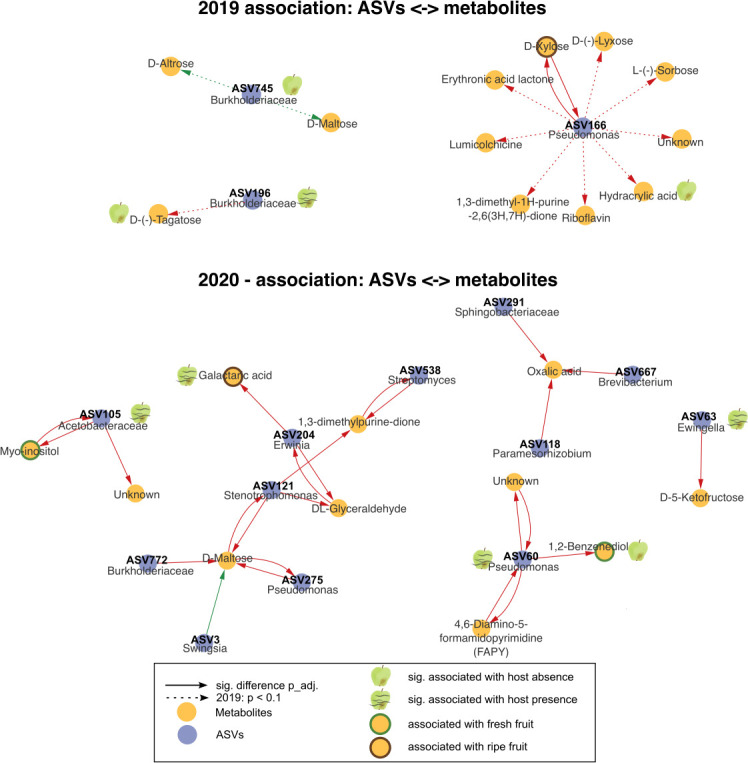
The relationship between individual bacterial ASVs and specific metabolites inferred from linear mixed-effects models. Networks of ASVs and metabolites for apples from 2019 and 2020 based on linear mixed-effects models (Metabolite~ASV and ASV~Metabolite). Sampling time point was included as random variable to account for dependencies of apples sampled together (details in Table S40). Green arrows indicate a positive intercept for the fixed effect, and red arrows a negative intercept for the fixed effect. Only significant associations after BH adjustments are shown, except dotted lines for year 2019, indicating associations with *P* < 0.1. Apples with or without worms correspond to ASVs or metabolites linked to host presence or absence via Deseq2 (Fig. 7C) or a linear mixed-effects model (Fig. 8; Table S39). The colors of metabolite node contours denote prior associations with fresh or ripe fruit.

Considering the above inferred association of ASVs and worm presence, we next wondered whether specific apple ASVs are connected to apple metabolome characteristics. Therefore, we assessed to what extent the abundance of certain ASVs on apples is associated with single metabolites from apples, as inferred from our metabolome analysis. Using a linear mixed-effects model analysis (see Materials and Methods), we identified more negative associations, indicated by the negative intercept of the fixed effect (Fig. 9; Table S40). In detail, in 2019, ASV166 (*Pseudomonas*) was negatively associated with multiple metabolites. One of these metabolites (hydracrylic acid) was associated with worm absence on apples. Moreover, D-xylose is significantly negatively associated with ASV166. However, neither the ASV nor the sugar is associated with the presence of worms. We further found a negative association between ASV 196 (also associated with worm presence) and D-(−)-tagatose (associated with worm absence). The data from 2020 produced a more complex network, indicating an intricate relationship between metabolites and ASVs, including one positive association between *Swingsia* and D-maltose.

Finally, we wondered in how far the whole apple microbiome can explain the different metabolite levels in apples. Using glmnet, we found that the identified compounds have an average relative root mean squared error (RMSE) of 0.22 (Table S41), suggesting that the ASV matrix predicts most compounds sufficiently well (average span of metabolites = 4.5, 0.99 average RMSE). The compounds with the lowest RMSE were beta-D-galactopyranoside (relative RMSE = 0.13) and 4-ketoglucose (relative RMSE = 0.05) for the respective years, and their levels are therefore very well predicted by the total ASV matrix.

The metabolome matrix can predict the abundance of different ASVs to a similar degree (average relative RMSE = 0.19 and 0.16 for 2019 and 2020, respectively) (Table S42). The lowest relative RMSE was identified in 2019 for ASV252, *Turicibacter* (ASV252, 0.07), and in 2020 for ASV100, *Aquabacterium* (ASV100, 0.074).

## DISCUSSION

In this study, we used *Caenorhabditis* nematodes and their apple source as a model to characterize the process of microbiome assembly in a host organism and its directly connected substrate. This direct host–substrate comparison revealed that the microbiomes of individual worms from the same substrate sample are both (i) highly similar (Fig. 3B) but also (ii) distinct, showing high individuality (i.e., highly individual, characterized by the presence of unique microbes; Fig. 3A). When comparing worm microbiome samples from different studies, sampled from a variety of substrate types, we found that (iii) worm microbiomes are largely substrate-specific (Fig. 4). Our analyses further suggest that (iv) worm microbiomes have a more significant influence on shaping the apple microbiome than vice versa (Fig. 5B and C). Moreover, our characterization of the substrate apple metabolomes identified (v) specific compounds that associate with worm populations in their natural habitat and are indicative of fruit ripening, including certain sugars and fatty acids (Fig. 8). Below, we first discuss differences and similarities among host and substrate microbiomes and how our observations enhance insights into the assembly process. Thereafter, we discuss our observations on host presence in relation to the apple micro- and metabolomes.

### The *Caenorhabditis* microbiomes are influenced by stochastic processes and strongly linked to its respective substrate

The worm “core” microbiome is context-dependent. *C. elegans* has been isolated from apples before (21, 35, 42, 43), and studies on the natural microbiome of *C. elegans* from diverse compost and fruit samples showed that certain bacterial taxa are more commonly found (13, 44), possibly suggesting that worm microbiomes are to a certain degree conserved. Our comparative analysis across several independent studies now demonstrates that worm microbiomes vary substantially, dependending on their respective substrate source (Fig. 4). For instance, members of the *Acetobacteriaceae* dominate worm microbiomes from apples in our current study, consistent with the previous analysis of worm-containing apples (42), but in contrast to studies that characterized worm microbiomes obtained from other substrate types such as compost material (13, 21).

Apple microbiomes are (functionally) more diverse than worm microbiomes, indicating higher specialization and competition in worm microbiomes (Fig. 2E). In detail, worm and apple microbiomes share the majority of abundant ASVs (Fig. 2A). *Caenorhabditis* nematodes feed on bacteria; hence, an overlap of source and host microbiome can be expected. However, worm and apple microbiomes differ in their alpha-diversity and overall community composition (Fig. 2B and C). Additionally, worm microbiomes have a smaller functional potential as inferred by PICRUSt2 (Fig. 2E). This aligns with the concept that, compared to apples, worm hosts represent a more closed and controlled ecosystem (34), which could favor microbial specialization. At the same time, competition for resources and space is likely to increase between microbes within such a closed ecosystem (45), as suggested for the *C. elegans* microbiome before (37, 46, 47) and supported by the larger number of negative edges in worm rather than apple microbiome co-occurrence networks (Fig. 2D).

Worm microbiome assembly is most likely driven by dispersal limitation (Fig. 5A) and potentially highly influenced by priority effects, as indicated by the observed inter-individual differences of worm microbiomes (Fig. 3) (13) and the results of the iCAMP analysis (Fig. 5A). First, this indicates that the composition of worm microbiomes is not fixed but rather shows some flexibility as to the presence of individual bacterial taxa, at least on the lower taxonomic levels. Indeed, previous work found that the overall characteristics of the *C. elegans* microbiome seem to be phylogenetically conserved (46), something that has been described before for other types of bacterial communities (as reviewed in reference 48). Moreover, most derived functions of the *C. elegans* microbiome were previously suggested to be ubiquitous, and only some are covered by few of the strains (6). Second, a high inter-individuality indicates that assembly processes are rather stochastic, as shown for *C. elegans* before (27), and influenced by priority effects (49). Indeed, we observed that worm and apple microbiome assembly is mostly driven by dispersal limitation (Fig. 5A), a highly, but not exclusively, stochastic process. This finding is consistent with that for mammalian microbiomes across the globe, which are strongly shaped by dispersal limitation due to physical distance, independent of diet and genotype (16). Since worms are not very mobile, strong inter-individuality may well be explained by physical distance between samples. Alternatively, and a lasting consequence of priority effects (25), the observed multiple states of worm microbiomes might represent different transient states that will eventually converge to the same state (50, 51) or compositional cycles, for instance due to the presence of predator–prey interactions (52–54), and indeed, the presence of bacterial predators belonging to the *Bdellovibrionota* in the *C. elegans* microbiome was shown before (13, 55).

Worm microbiomes shape apple microbiomes to a stronger extent than vice versa (Fig. 5B and C). Our results using FEAST and machine learning indicate that worm microbiomes influence the composition of apple microbiomes at least partially, possibly indicating a kind of niche construction (56–58) by the nematodes, which may be favored by selection as it can contribute to higher reproductive rates and thus evolutionary fitness of the worms. At the same time, we observed that worm microbiomes are to a certain extent substrate-specific, indicating a more complex interaction between source and sink.

Overall, our data suggest an intricate relationship between worm and substrate microbiomes: Initially, the worms recruit their microbes from the microbial community present in the substrate, most likely influenced by stochastic processes, including priority effects. Our data then suggest that, as a next step, the nematodes and their microbiomes do influence the microbiome composition of their substrates, possibly by defecation and shedding of nematode-associated microbes into the substrate environment and/or by releasing specific metabolites into the substrate that directly affect the presence of specific microbes and/or influence the substrate environment in a specific way that in turn affects microbial presence.

### Signals of fruit ripening and decay are associated with worm presence

Biotic and abiotic factors differentiate worm-free and worm-harboring apples, indicating that worm-harboring apples are riper, thus representing a habitat that allows for and is shaped by a specific microbial community. We identified pH as an important abiotic factor that correlates with worm presence on apples (Fig. 6A) and is known to influence microbial communities (59, 60). In line with this, apples harboring worms have a significantly different microbiome (Fig. 7).

Additionally, we observed a trend for a negative correlation between temperature and *Caenorhabditis* prevalence on apples (Fig. 6B), consistent with a previous field study (35). Moreover, we found most *Caenorhabditis* nematodes on apples with pH values between 3 and 5, consistent with previous laboratory findings, which showed that *C. elegans* can survive a pH between 3.1 and 11.9 (61). In contrast, the presence of worms on apples was not influenced by average and minimum temperature, precipitation, and barometric pressure of the environment. This is in contrast to a study (35) reporting that rain was positively correlated with *C. elegans* occurrence in compost samples. However, the latter study was based on 1.5 years of continuous sampling, thereby including data from spring and early summer, which likely explains the different findings. Additionally, we found that apple pH increases over sampling time (Fig. S10A) indicating that the apples are ripening (62, 63).

Genes and metabolic pathways inferred for the microbial taxa of the apple microbiomes via PICRUSt2 suggest that apples harboring *Caenorhabditis* worms are riper than those without nematodes (Fig. 7E). Many of the pathways enriched in worm-containing apple microbiomes contain 1-deoxy-D-xylulose-5-phosphate as an intermediate or product. Xylulose-5-phosphate is produced when xylose is metabolized via the isomerase pathway to xylulose, which in turn has previously been associated with cell wall disassembly in pitaya fruit (64), indicating fruit ripening. Using PICRUSt2 predictions, we further identified the D-galacterate degradation pathway to be significantly more abundant in apple microbiomes with worms (Fig. 7E). Our metabolomic measurements confirmed that galactaric acid (= galactarate) is more abundant in these apples (Fig. 8B). Importantly, galacturonic acid can be reduced and oxidized to D-galactarate during the degradation of pectin. Pectins and, hence, galacturonic acid are known to be present in high amounts in apple cell walls (65), and the increase in genes associated with its breakdown indicates ripening or decay (66).

Consistent with these findings, our metabolomic analysis of apples also demonstrates a significantly higher prevalence of *Caenorhabditis* nematodes on apples with metabolic signatures of ripe fruits (Fig. 8). In detail, fruit ripening induces several physiological (67) and metabolic (68) changes, including cell wall remodeling (69, 70), cuticle biosynthesis (71), and increase in the fruit pH (67, 72), the latter consistent with our observed correlation between presence of worms and high apple pH (Fig. 6A). Further, soluble sugar accumulation is an integral part of the ripening process. For instance monosaccharides, like arabinose, have been associated with cell wall disassembly of pitaya fruit before (64), and our measurements identified higher amounts of arabinose in worm-associated apples. We also observed higher gluconic acid amounts, which was found in previous work to correlate positively with the ripening stage of pineapples (73), infections in apples (67, 74), and cell wall disintegration (75, 76).

Additionally, several of the metabolites enriched in worm-free apples have been associated with fresh fruits before: malic, citric, and tartaric acids are the predominant fruit acids in cultivated apples and wild apple species (77). Additionally, malic acid has been associated with fresh strawberries (78). Another example is benzenediol, also known as catechol, with higher amounts of benzenediol being related to fresh, non-brown fruits (79). Furthermore, galactose has been shown to decrease in ripe pineapples (73) and other fruits (80, 81).

### Apple ripeness is associated with worm proliferation potentially by providing beneficial microbes and metabolites

An association of *C. elegans* with decaying apples was reported previously (42) and suggested to result from a higher bacterial load of rotten apples. We now identified bacteria previously shown to be beneficial for *C. elegans*, i.e., *Gluconobacter* (42), to be highly prevalent in worm microbiomes, indicating that their growth was promoted on worm-harboring apples.

Certain apple microbiome members appear to shape the ripening of fruits. Our ASV-metabolite network indicates mostly negative associations between apple microbiome members and apple compounds (Fig. 9). One example refers to xylose metabolism. Xylose is a monomer of xylan, which is an integral part of the plant cell wall and disassembled during ripening. Interestingly, xylose correlates negatively with *Pseudomonas* abundance, possibly suggesting a direct or indirect relationship. Myo-inositol and ASV105 (belonging to the *Acetocbacteraceae*) abundances are another example of a negative correlation. Myo-inositol was previously shown to contribute to the regulation of osmotic pressure in blueberry fruits, thereby maintaining turgor and fruit firmness (29). Moreover, it was negatively correlated with the ripening process of pineapples (73). In line with this, bacteria of the *Acetobacteraceae* are associated with the fermentation of sugars to acetic acid, a process occurring during vinegar production.

Using machine learning, we identified specific metabolites whose abundance can be explained by the bacterial ASV matrix of the apples (Table S41). Here, beta-D-galactopyranoside and 4-ketoglucose were the best explained compounds for 2019 and 2020, respectively. Both compounds are part of the galactose and glucose metabolism, respectively, which represent the main sugars found in apples. The ability to use these sugars or products of their degradation might be a major advantage for bacteria inhabiting apples. Conversely, the abundance of *Turicibacter* (2019) and *Aquabacterium* (2020) bacteria are best explained by the apple metabolite matrix (Table S42). Indeed, *Turicibacter* was previously associated with apple diet in mice (82) and as potential modulator of host fat metabolism (83) and might therefore represent a common microbe on fruit and in associated hosts. To our knowledge, *Aquabacterium* has not yet been associated with fruit ripening or decay. However, this bacterium was reported to metabolize organic acids, which are also prevalent in apples, but not any carbohydrates (84). Its possible involvement in the fruit ripening process warrants further analysis.

### Conclusions

Overall, our study elucidates the complex relationship between a host microbiome and its directly connected substrate microbiome, using *Caenorhabditis* nematodes and its apple substrate as a model. Our analyses strongly suggest that the nematode microbiome community has a more substantial influence on shaping the microbiome and, thereby, the metabolic capacity of its apple source than vice versa. We conclude that host-associated bacterial communities significantly impact their environment, be it the worm microbiome influencing its environment (the apple microbiome) or the apple microbiome influencing the fruit’s metabolome. Our study further revealed a strong impact of stochasticity on the assembly process and identified individual bacterial ASVs and metabolites that are significantly associated with proliferating hosts.

## MATERIALS AND METHODS

### Sampling and sample preparation

All apple samples were collected from a compost heap of the botanical garden in Kiel (coordinates: 54.34854, 10.11681) in 2019 and 2020. The apples came from an orchard in Kiel (coordinates: 54.29226, 10.10977). They were rinsed in water and evenly distributed onto the compost heap. After an initial 3-week period, we started weekly sampling of 10 apples over a period of 11 (start: 5 September 2019, apples were added on 14 August 2019) and 9 weeks (start: 8 September 2020, apples were added on 18 August 2020), respectively. At each sampling time points, apples were selected randomly for sampling, with a preference for apples with the highest grade of decay, because *Caenorhabditis* nematodes are known to be most common in highly decayed apples at this location (35). The selected apples were removed from the compost heap, placed in a sterile freezer bag, and brought to the laboratory. Pieces of apples (approx. 10–20 g of the most decayed region) were used for bacterial DNA extraction and apple metabolomics and stored at −80°C. For the remaining apple material, the pH was measured using indicator sticks (pH-Fix 0-14, Macherey–Nagel), while abiotic weather data were derived from the weather station in Kiel Holtenau (54.3833, 10.15) and downloaded from meteostat.net (meteostat ID 10046). For isolation of nematodes, we used additional pieces of the apples and spread them evenly onto a 9 cm petri dish, covered with viscous (V) medium containing 1.2% hydroxypropyl-methyl cellulose (HPMC) (85). This allows for the worms to float to the top, which simplifies the collection of worms with a pipette. We randomly collected as many nematodes as possible, although usually not more than 20. Nematodes were washed three times in M9-T (M9 with 0.05% Triton X-100) for 5 min each in order to sufficiently remove cuticule-associated microbes, and subsequently used for DNA isolation, followed by PCR-based species identification and 16S amplicon sequencing of the microbiome.

### DNA extraction from worms

Single-washed worms were collected in single wells of 96-well plates containing 1–3 1 mm zirconium beads and 10 µL 2× Tris EDTA buffer, pH 8 with 1 mg µL^−1^ proteinase K. These 96-well plates were used for DNA isolation by first freezing at −80°C for at least 16 h, shredding in the Geno/Grinder (Spex Certiprep 2000) for two times for 3 min at 1,500 strokes ^−1^min^−1^, and subsequent proteinase K digestion (1 h 55°C, 20 min 98°C), as described earlier (13, 21, 86). Worm identity was checked using diagnostic PCRs as previously described (13, 21, 86). In detail, worm DNA was amplified using *C. elegans*- (nlp30-F and nlp30-R [35]), *C. briggsae*- (Cbriggsae-F and Cbriggsae-R [87]), and *C. remanei*-specific (Cre-ITS2-F1 and Cre-ITS2-R4 [35]) primers. Only worms with a positive PCR signal for one of these primers were further processed and claimed “*Caenorhabditis*-positive.” We focused on these three taxa because in our previous work, we never identified any other *Caenorhabditis* species at this location (13, 21, 35, 88).

### DNA extraction from apples

Frozen apple pieces were homogenized using the bead ruptor 96-well plate homogenizer (Omni International). DNA was extracted using the innuPREP Plant kit (Analytic Jena) following the manufacturer’s extraction protocol. In detail, 130–140 mg apple sample was added to the innuSPEED Lysis Tube E including 10× Keramik-beads (2.4–2.8 mm) and homogenized for 3 min at 25 Freq. The extraction was done with the Lysis solution OPT buffer. Finally, DNA was cleaned with the Genomic DNA Clean and Concentrator kit (Zymo Research) and dissolved in 50 µL elution buffer.

### Apple metabolomics

For the apple metabolomics, we adjusted a protocol from Fiehn 2016 (89). In detail, frozen apple pieces were homogenized in 2 mL screw cap tubes using the bead ruptor 96-well plate homogenizer (Omni International) for 3 min at 25 Freq and a mix of 2.7 mm soda lime glass beads (Carl Roth N032.1) and 2.3 mm zirconia/glass beads (Biospec Products, 5–6 beads in total). Exactly 100 mg homogenized apple was added to a new 2 mL screw cap tube and extracted in extraction solution 1, consisting of acetonitrile:isopropanol:water (3:3:2, vol:vol:vol), where acetonitrile and isopropanol had a neutral pH, and oxygen was removed with a vacuum pump. To each sample, 1 mL extraction solution 1 and 10 µL heliotrine (10 mg/mL) were added as internal standard. After 10 s of vortexing and shaking for 5 min at 4°C, samples were centrifuged for 2 min at 14,000 rcf. From this solution, 450 µL supernatant was evaporated at room temperature with a dried air flow using the Reacti-Vap Evaporator (Thermo Fisher Scientific). The samples were resuspended in 450 µL of extraction solution 2 (acetonitrile:water, 50:50, vol:vol, oxygen removed), centrifuged for 2 min at 14,000 rcf, and 300 µL of the supernatant was transferred to glass vials with inserts to allow complete drying using the Reacti-Vap. For the derivatization, 10 mL MOX solution (2% solution of methoxyamine-hydrogen chloride in pyridine; Thermo Fisher Scientific) was added, and samples were shaken at 30°C for 1.5 h. Finally, 91 µL MSTFA (Merck) and 2 µL alkane standard (C10-C40 all even; Merck) were added, mixed, and shaken for 0.5 h at 37°C. Those samples were submitted to gas chromatography (GC). Per sample, 1 µL was injected into the TSQ Duo GC-MS (ThermoFisher Scientific, Waltham), equipped with a Split/Splitless Injector at 280°C. The split ratio was 1:25, and the helium column flow was set to 1.2 mL/min. A TG-5SilMS (30 m × 0.25 mm × 0.25 µm) GC column was used with an oven program as follows: 50°C initial temperature for 5 min, followed by a temperature increase by 5.5 °C/min up to 300°C, which was maintained for 5 min. The mass spectrometry (MS) transfer line was set to 280°C, and the ion source was set to 300°C. Electron impact ionization was performed with 70 eV electron energy, and a full scan was recorded from 50 to 650 *m*/*z*. The raw data were afterwards deconvoluted using Compound Discoverer 3.1, and annotation of metabolites was performed based on the spectra comparison with NIST, Golm Metabolome Database (90).

### 16S sequencing

All information on used programs, their version, and parameters can be found in Table 1. The V3–V4 region of the 16S rRNA gene of apple (derived from 130 to 140 mg apple) and worm microbiome samples (derived from single worms) were amplified and sequenced with the Illumina Miseq platform and the v3, 2 × 300 bp Kit from Illumina using the primers 341F (CCTACGGGNGGCWGCAG) and 806R (GACTACHVGGGTATCTAATCC). Primers were trimmed from the raw reads using cutadapt (91). The reads were submitted to the Dada2 pipeline (92), allowing pseudo pool since data from two independent runs were combined in the analysis. The taxonomic annotation was done per sequencing run using the GTDB86 (GTDB_bac_ssu_r86_dada2) database and the Bayesian algorithm. The resulting sequence data were analyzed using R (93) and the package phyloseq (94).

**TABLE 1:** Analysis programs and parameters used

Program	Version	Parameters
R	4.2.1	
Dada2	1.1	truncLen was set to 260 and 200, allowing pseudo pool
		study comp: filter and trimming parameters: maxN = 0, maxEE = c(0.25, 0.25), truncQ = 2
Phyloseq	1.44.0	
Deseq2	1.38.3	alpha = 0.01
Blast	2.10.1+	perc_identity 99 -qcov_hsp_perc 95
Glmnet	4.1–8	
Picrust2	2.5.2	Default settings
Lme4	1.1–34	
iCAMP	1.5.12	Rand.time =1000
		ds = 0.2
		abcut = 3
		icres = iCAMP::icamp.big (ds = 0.2, pd.cut = NA, sp.check = TRUE, phylo.rand.scale = "within.bin", taxa.rand.scale = "across.all", phylo.metric = "bMPD", bin.size.limit = 24, detail.null = FALSE, ignore.zero = TRUE, correct.special = TRUE, unit.sum = rowSums(comm), special.method = "depend", ses.cut = 1.96, rc.cut = 0.95, conf.cut = 0.975, omit.option = "no",meta.ab =NULL)
		icbin = iCAMP::icamp.bins(icamp.detail = icres$detail,treat = treatment, clas = clas,silent = FALSE, boot = TRUE, rand.time = rand.time,between.group =TRUE)
NST	3.1.10	pnstout = NST::pNST(abundance.weighted = TRUE, phylo.shuffle = TRUE, output.rand = TRUE, SES = FALSE, RC = FALSE)
Ampvis2	2.7.33	cut_a = 0.1 (Abundance cutoff in percent), cut_f = 0.1 (Frequency cutoff in percent)
Mia	1.9.12	
vegan	2.6–4	
Conet	1.1.1	Allow explore links between higher-level taxa and parent-child exclusion
		Minocc = 20, col normalization
		Pearson, Spearman correlation, Mutual exclusion
		Bray-Curtis and Kullback-Leibler dissimilarity distances
		Thresholds: edge number 1000, top and bottom
		Randomization and renormalization based on edge scores,
		Bootstrapping using brown for p-value merging, filter unstable edges, benjaminihochberg for multiple testing correction, p-value threshold: 0.05
Cytoscape	3.9.1	
indicspecies	1.7.14	Defaul settings
metabolomicsR	1.0.0	“log” and “scale” transformation
factoextra	1.0.7	
ggplot2, ggcorrplot, ggpubr	3.5.1	
	0.1.4.1	
	0.6.0	
cutadapt	1.18	
sina	1.6.1	
picante	1.8.2	
ggpicrust2	3.4.4	ALDEx2 and Wilcoxon rank test
Matchit	4.5.5	Method = exact
lmerTest	3.1–3	
ape	5.8	
FEAST	0.1	different_sources_flag = 0

### 16S data analysis and statistics

Sequence data were analyzed with the phyloseq package. Chloroplast and mitochondrial reads were removed. We applied a filtering step to retain only ASVs that met three criteria: (i) a prevalence of at least five samples, (ii) a total abundance of at least 10 across all samples, and (iii) a minimum abundance of at least five in any individual sample. Unless otherwise stated, we used count data for the analyses. Information on used R packages are given in Table 1.

Alpha-diversity was either determined using Chao1 (estimate of species richness that incorporates the likely presence of undetected species due to the compositional data of amplicon sequence data), Simpson diversity (estimate of diversity that accounts species richness and evenness, with more emphasis on dominant species), or Shannon diversity (estimate of diversity that accounts species richness and evenness, with more emphasis on rare species) and using the respective function from the vegan package (95).

Statistical analysis using linear mixed models was performed with the package lme4 (96). Such linear mixed models were used to account for dependencies among samples because multiple worms were isolated from single apples, and several apples (and their worms) were collected at the same time point. These dependencies defined by apple and sampling time point were considered as random factor in the model. lmerTest (97) and stargazer (98) were used for subsequent analysis of the linear mixed models.

Beta-diversity was either calculated using the Aitchison distance or the unweighted UniFrac distance. In detail, the latter incorporates phylogenetic information. In contrast, the Aitchison distance is the Euclidean distance of centered log-ratio (clr) transformed data and was proposed by Aitchison et al. (99) and is particularly suited for compositional data (100). Given the clr transformation and Euclidean geometry of the data, a PCA is better suited than a PCoA to explore the variance of such data (100). Statistical testing on beta-diversity data was performed using PERMANOVAs, and more specifically by using the adonis2 function of the vegan package. The argument “strata” was used to account for dependencies between data, for instance to consider the cases, when multiple worms were isolated from the same apple or when multiple apples were collected at the same sampling time point. Multiple testing was correcting by adjusting *P*-values using Benjamini and Hochberg—BH (101), when necessary.

Venn diagrams were generated with the ampvis package (102). Core ASVs were defined as ASVs that appeared in at least 80% of the samples of a respective group of samples (i.e., worms 2019, worms 2020, apples 2019, apples 2020, etc.). Indicator ASVs were identified with the indicspecies package (103) using the multipatt method and 999 permutations. This method takes two values into account. Value A is the specificity, i.e., how exclusively an ASV is associated with a particular group. Value B gives the fidelity, i.e., how frequently does the ASV occur within the group.

Picante (104) was used to calculate Faith’s phylogenetic diversity (105). Matchit (106, 107) was used to specifically compare Faith’s phylogenetic diversity of apple and worm samples with matching Chao1 values. To test if apple or worm microbiomes, respectively, have a higher or lower phylogenetic diversity than expected by chance, we computed null distributions for each apple and worm microbiomes separately. For this, we first calculated the means for PD and Chao1 for worms that were isolated from the same apple to exclude these dependencies between the worm samples from the analysis. We then randomly drew worm and apple samples that had a Chao1 value within the specified range for worms. Thus, we used the observed range of worm Chao1 values as a cut off for randomly picking the worm and apple samples to generate a new “worm” null distribution. Likewise, we used the range of observed apple Chao1 values as a cut off to draw worm and apple samples to generate a new “apple” null distribution. Thereafter, we calculated how likely the observed PD means for apple and worm microbiomes are under these null distributions. The same was done for EC and MetaCyc pathway richness.

DeSeq2 (108) was used to identify differentially abundant ASVs on geometric means of count data. Zeros were ignored.

Co-occurrence networks were generated using conet (109). More details on the specific parameters are given in Table 1. Cytoscape (110) was used to graphically illustrate networks.

PICRUSt2 (111) was used to infer genetically encoded functions based on the 16S abundance data using the standard parameters and the MetaCyc pathway database (112). Ggpicrust2 (113) was used to infer statistical differences in metaCyc pathway (112) abundances between microbiomes from apples with and without worms using ALDEx2 (114–116) and a Wilcoxon rank test to identify statistical significance. All *P*-values were adjusted with BH.

To infer processes that influenced the assembly of the worm and apple communities, NST (117, 118) and iCAMP (117) were used on count data. We used the package “ape” (119) to construct rooted phylogenetic trees for both methods. We then followed the instructions of the manuals. In detail, for iCAMP, we defined “environment” (i.e., apple and worm) and “sampling time point” as treatments (and thereby as grouping factors) and performed the bootstrapping on the variable “environment.” We used the following data as environmental data: pH, average, minimum, and maximum temperature of the environment, precipitation, wind speed, peak gust, barometric pressure, and weeks on compost. We assessed niche differences and within-bin phylogenetic signal. We used the same data to calculate the pNST and again using “environment” for bootstrapping.

Glmnet (120) and FEAST (41) were used to evaluate source-sink dynamics. For FEAST, we used the count data of ASVs that appeared at least three times in at least 10% of the samples as input and considered the microbiomes of worms and their source apple. We treated the apple microbiome as the source and the microbiomes of all worms within it as the sink or considered all worm microbiomes as the source and the directly connected apple microbiome as the sink. We did this separately for each apple. From this, we were able to assess for our entire data set to what extent the apple microbiome samples influence the composition of directly connected worm microbiome samples and also vice versa. For the glmnet approach, we used again the relative abundances of ASVs that appeared at least three times in at least 10% of the samples and randomly associated 100 true individual worm–source apple pairs (100 times 19 pairs) for each apple. We did this since multiple worms were usually collected from a single apple. We then predicted the abundance of each non-zero ASV (total ASVs: 328, number of non-zero ASVs is different for each pairing, around 200 ASVs) present in apples (worms) by all the worm (apple) ASV abundances. The prediction was done by a penalized regression model (lasso) from the glmnet package. We checked the accuracy of the prediction by prediction error (RSME) and explained variance (R2). In total, there were 100 runs for each non-zero ASV (<328) that result in >20,000 predictions. In order to account for data inflation, we calculated the mean RSME and mean R2 for each ASV, indicating how well the ASV could be predicted.

In order to compare the resulting sequencing variants with the established extended CeMbio resource (37, 121) we used blast (122) and compared the 16S data with a database containing only the 16S data of the 43 CeMbio strains. We only considered those sequences that 100% match the CeMbio 16S sequence.

### Comparison with previous studies

In order to compare the results of this study with the results of the former studies by Dirksen et al. (21) and Johnke et al. (13), samples were re-analyzed together as done previously (13). In detail, primers were trimmed using cutadapt (91). The dada2 (92) pipeline was run for samples from the different studies separately. Sequences were then prealigned using sina (123), subsequently truncated to the same 16S region to account for different 16 sequencing protocols (the V4 region), and, finally, ASVs were taxonomically assigned using the SILVA_v138 database. Chloroplast and mitochondrial reads were removed. Thereafter, sequences were analyzed with phyloseq (94).

### Metabolomics data analysis

Metabolomics data were ratio normalized using the added standard indizine. Samples were log transformed and scaled using the transformation function of the metabolomicsR package (124). To infer statistically significant differences of metabolite abundance between apples with and without worms, a linear mixed-effects model (using the lmer function of the lme4 package [96]) was used with the sampling time point as random factor to account for dependencies between apples that were sampled at the same time and pH as additional explanatory variable that we found in our above analysis to be significantly associated with the presence of nematodes. The resulting *P*-values were adjusted with BH. The metabolomes of apples with and without worms were further analyzed in a PCA using the R package factoextra (125). Further, a linear mixed model was used to (i) infer the effect of the sample community members (each ASV) and the pH (using the sampling day as random effect) on each metabolite of a sample using the lmer function of the lme4 package (i.e., Metabolite ~ASV + pH + (1|Sampling time point)). The same was done for the other direction (ii), i.e., ASV ~ Metabolite + pH + (1|Sampling time point). Again, the random variable “sampling time point” was included to account for dependencies of apples from the same sampling time point. For this, only ASVs that appeared at least three times in at least 10% of the samples were used using a centered log ratio ASV matrix as well as the log transformed and scaled metabolomics data. The resulting *P*-values were adjusted with BH. Glmnet (120) was used to (i) infer single metabolite data from the centered log ratio transformed ASV matrix and to (ii) infer single ASV abundances from the log normalized and scaled metabolomics matrix using the best alpha as calculated with the cvfit$lambda.min command. In both cases, only ASVs that appeared at least three times in at least 10% of the samples were considered.

## Data Availability

Sequences are publicly available at the SRA under accession number PRJNA1096453.

## SUPPLEMENTAL MATERIAL

Supplemental Figures msystems.01533-24-s0001.pdf Fig. S1 to S11.

Supplemental Tables msystems.01533-24-s0002.xlsx Tables S1 to S42.

ASM does not own the copyrights to Supplemental Material that may be linked to, or accessed through, an article. The authors have granted ASM a non-exclusive, world-wide license to publish the Supplemental Material files. Please contact the corresponding author directly for reuse.
