# Supplementary material for: Caenorhabditis nematodes influence microbiome and metabolome characteristics of their natural apple substrates over time
Source: mSystems. 2025 Jan 10;10(2):e01533-24. doi: 10.1128/msystems.01533-24 (PMC11834410; doi:10.1128/msystems.01533-24)
Supplement: Supplemental Figures — Fig. S1 to S11. [file msystems.01533-24-s0001.pdf]

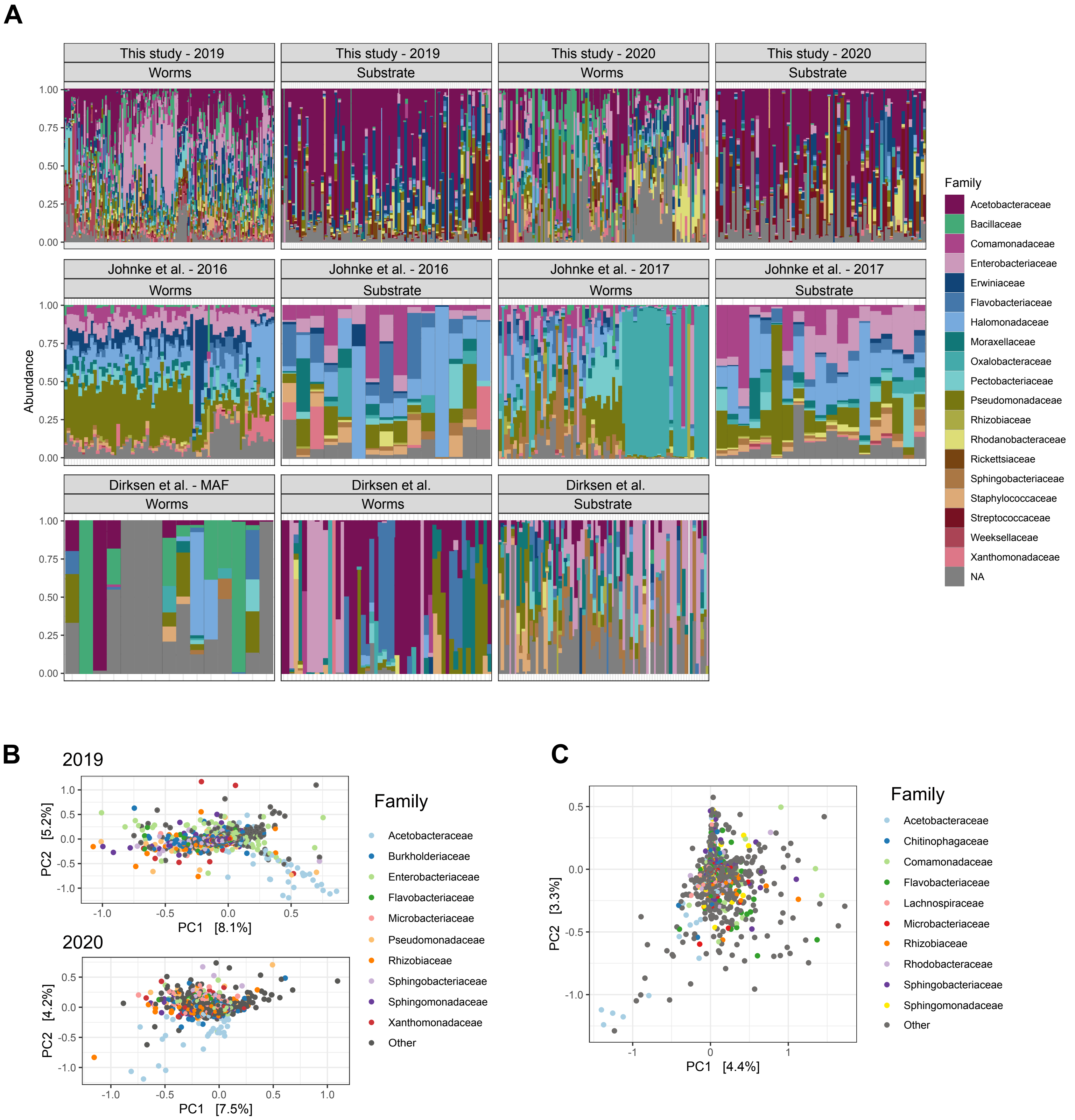

Figure S1: Community composition of *Caenorhabditis* worm and substrate microbiomes. A: Bar plots of community composition per sample, ordered according to sampling time point of samples from this and previous related studies. Family identity is denoted by different colors. Only taxa that were present in more than 20% of samples with an abundance great-er than 3 were considered. B: Beta-diversity plots using Aitchison distances corresponding to Fig. 2C, but showing taxa instead of samples. Here, the distribution of taxa is based on their relative abundance and diversity across samples. Separately clustering families were more abundant in certain samples. Family identity is denoted by different colors. Only the ten most abundant families were considered for the analysis. C: Beta-diversity plots using Aitchison distances corresponding to Fig. 4A, but showing taxa instead of samples. Here, the distribution of taxa is based on their relative abundance and diversity across samples. Separately clustering families were more abundant in certain samples. Family identity is denoted by different colors. Only the ten most abundant families were considered for the analysis.

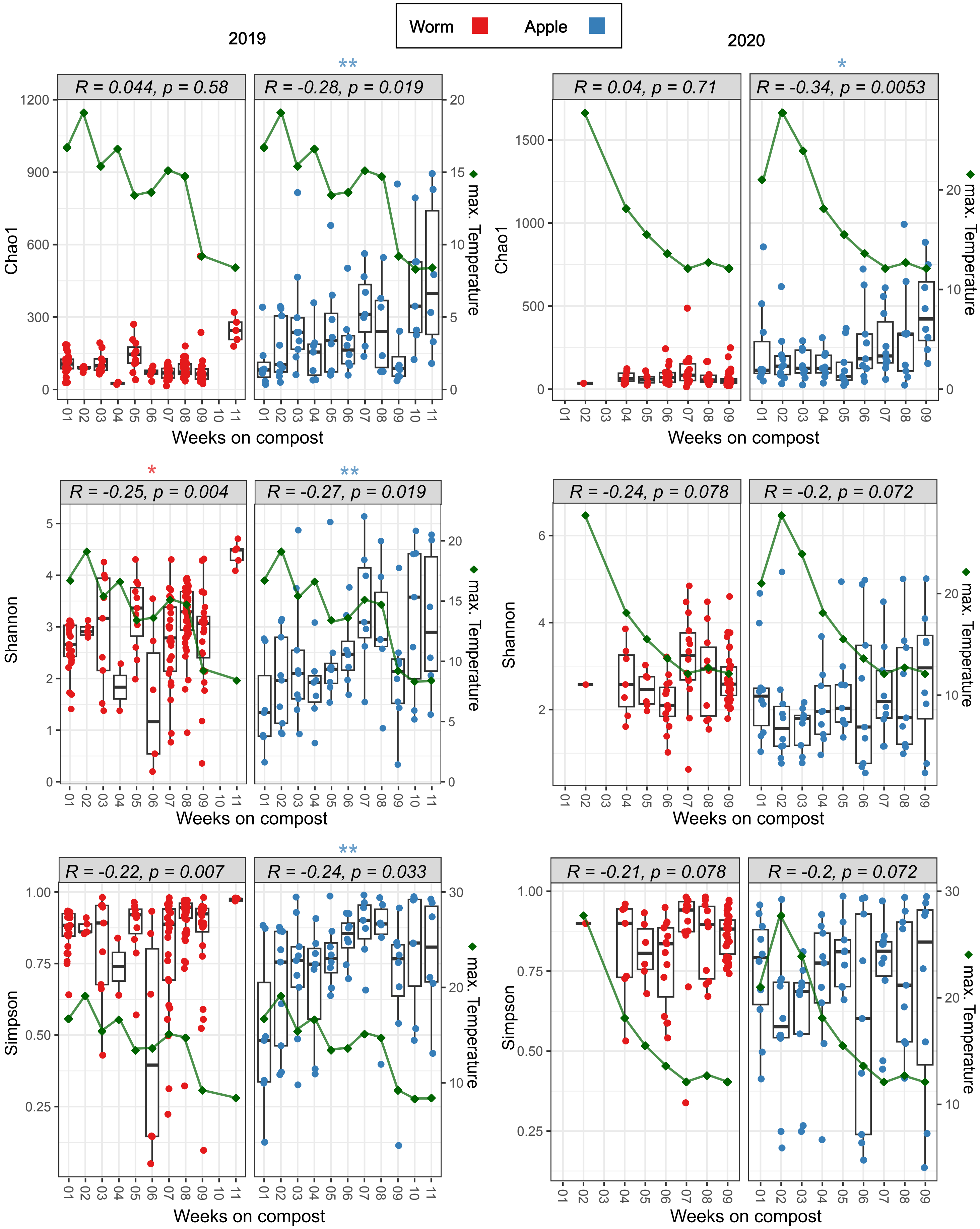

Figure S2: Variation in alpha-diversity of worm (red) and apple microbiomes (blue) per sampling time point (i.e. weeks on compost) in 2019 and 2020. Green diamonds indicate the maximum temperature of the respective sampling time point. Results of the Spearman correlation between max. temperature and alpha-diversity are given in the grey blocks. P-values were BH adjusted. The relationship between weeks on compost and alpha diversity was assessed with a linear mixed-effects model using alpha diversity as response variable and weeks on compost of apples as fixed effect. To account for dependencies of worms that were isolated from the sample apple, apple ID was used as random factor. Transfor-mations of diversity indices were performed when necessary to ensure normality of residu-als. All p-values were corrected using BH. Significant associations are indicated by asterisks above panels. Further details are given in Table S5.

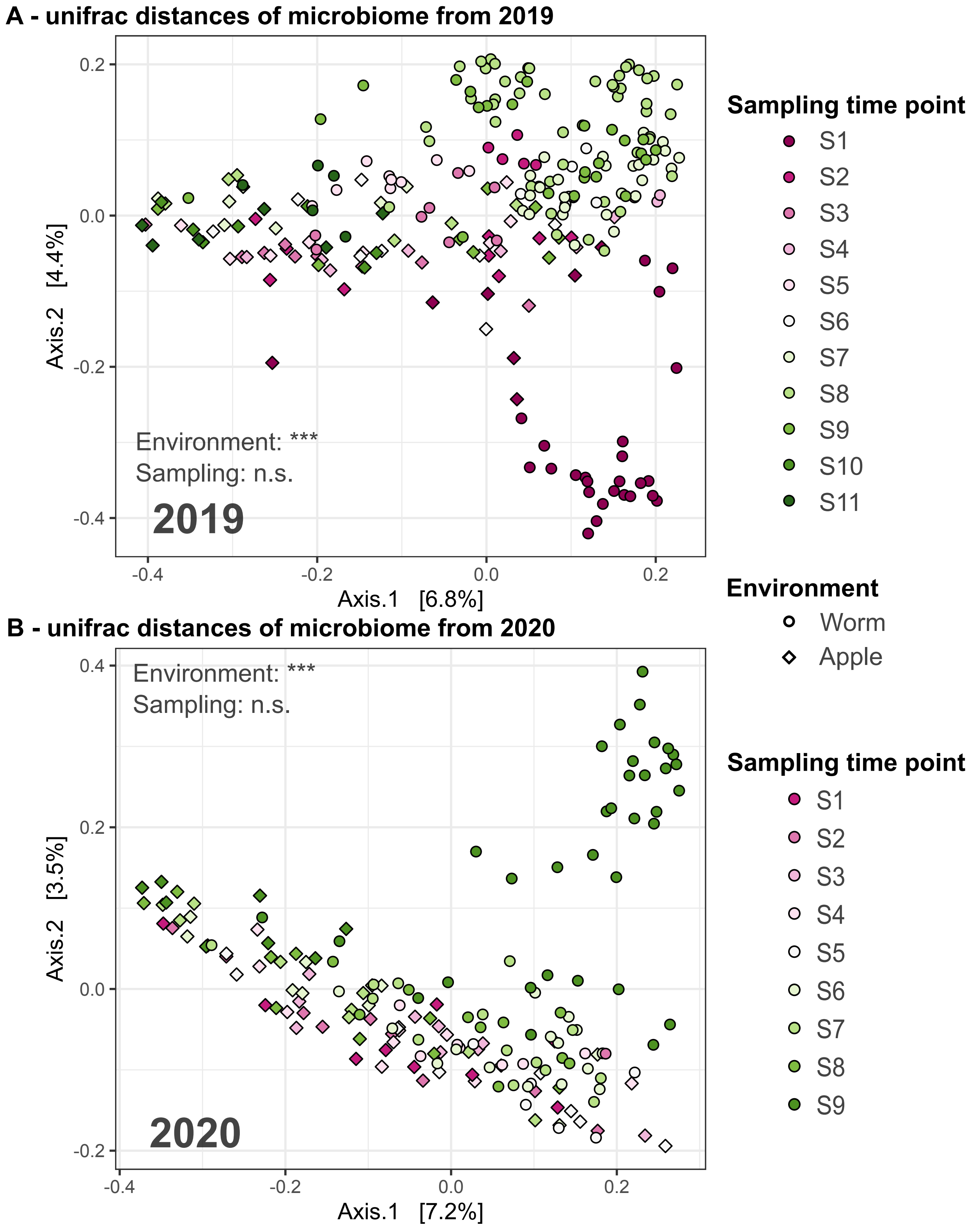

Figure S3: Beta-diversity shown as unweighted UniFrac distance between microbiome samples from 2019 (A) and 2020 (B). Colors denote the different sampling time points. Each shape represents a sample, while circles and diamonds indicate the microbiomes of *Caneorhabditis* worms and apples, respectively. Statistics were performed using adonis2 and the formula UniFrac distance ~ Environment or sampling time point, with the specific apple for each sampling time point as random factor (strata) to account for dependencies of worms that were isolated from the same apple. Further details on the statistics are given in Table S7.

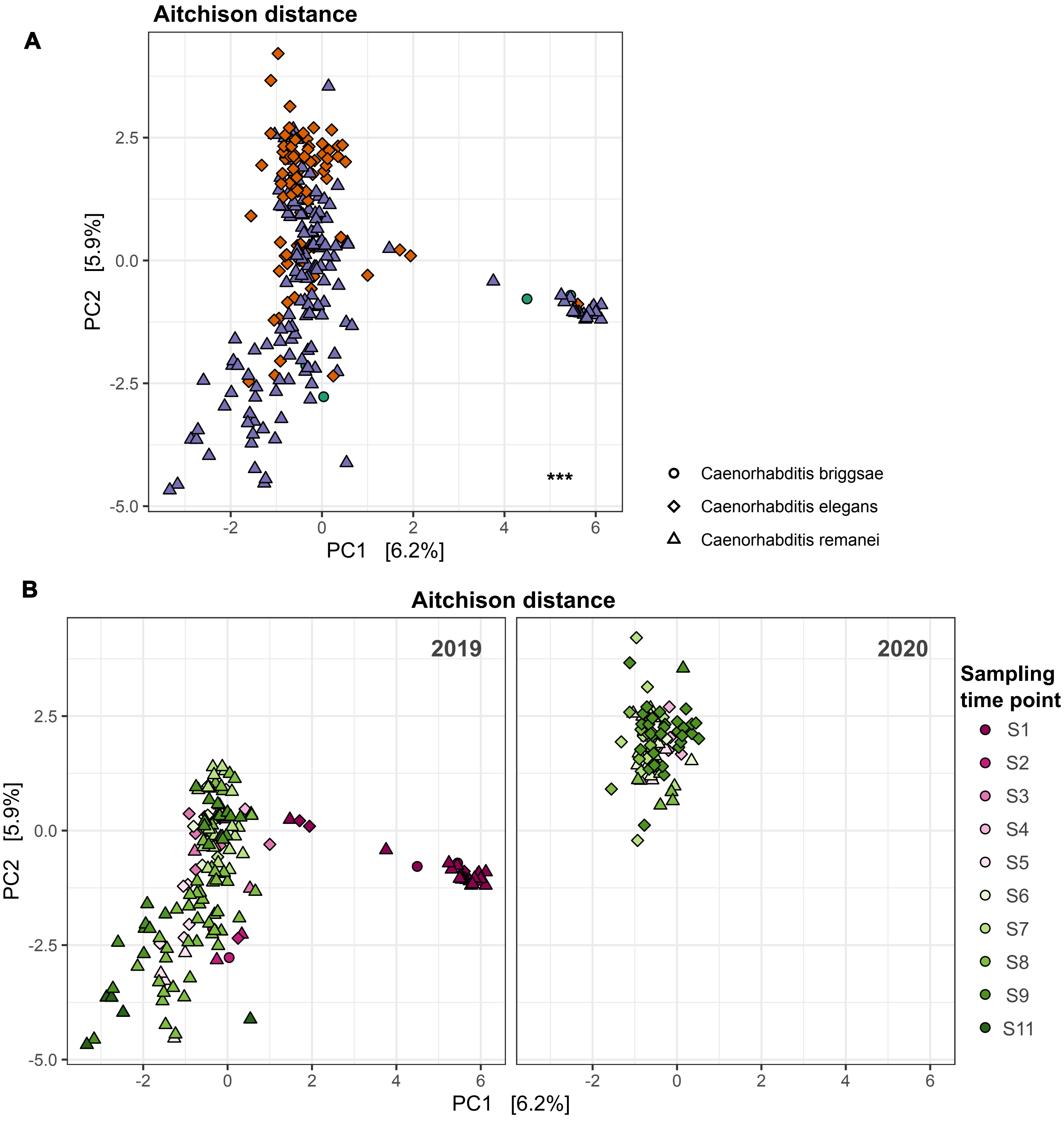

Figure S4: Beta-diversity shown as Aitchison distance between any two samples of *Caeno-rhabditis* worm microbiomes. Colors reflect different *Caenorhabditis* species (A) and differ-ent sampling time points (B). Shapes indicate worm species. Samples are separately shown per year in B, indicating that variation in microbiome composition is mainly driven by sam-pling year. Statistics were performed using the adonis2 function of the vegan package and the formula Aitchison distance ~ Host species with the specific apple ID per sampling time point as random factor to account for dependencies of worms sampled from the same ap-ple and apples sampled at the same time point. Further details on the statistics are given in Table S8.

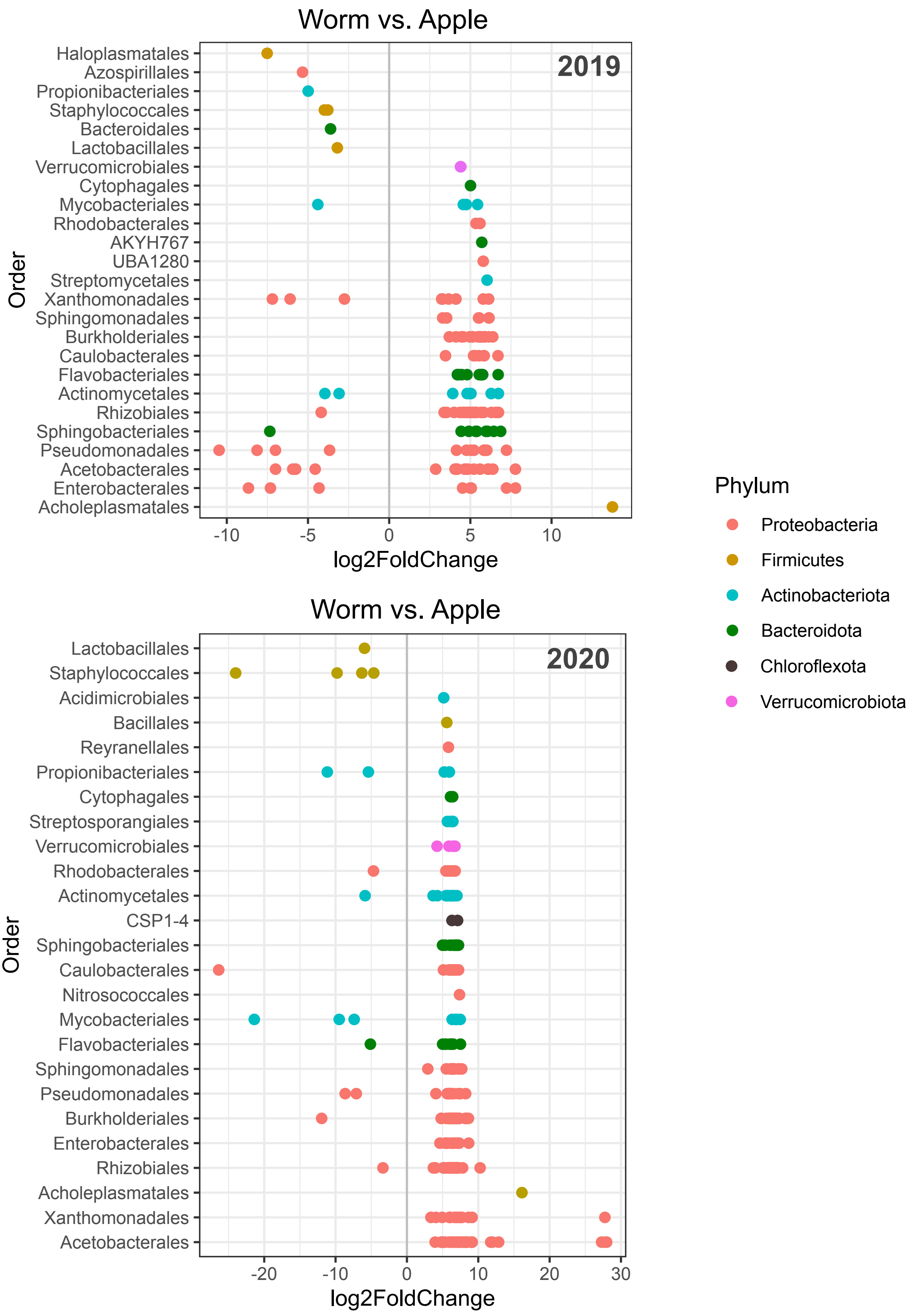

Figure S5: Significant differentially abundant ASVs between *Caenorhabditis* worm and ap-ple samples according to DeSeq2 in 2019 and 2020. Colors indicate the phylum of each ASV. ASVs on the left side ("Worm") are significantly more abundant in worms, while those on the right ("Apple") are significantly more abundant in apples.

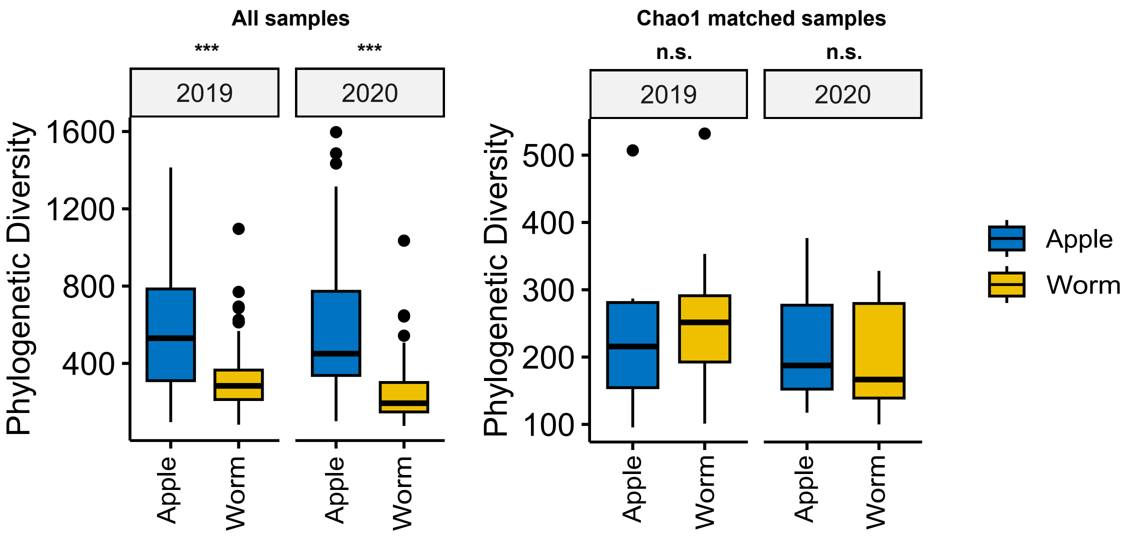

Figure S6: Faith's phylogenetic diversity between *Caenorhabditis* worm (yellow) and apple microbiomes (blue) in 2019 and 2020 using either all data or only worm and apple data with exactly matching Chao1 values. Statistics were performed using a linear mixed-effects model and the formula phylogenetic distance ~ Environment (i.e., worm or apple) + pH with the specific apple for each sampling time point as random factor. Further details on the statistics, including p-values for the term "pH" are given in Table S13.

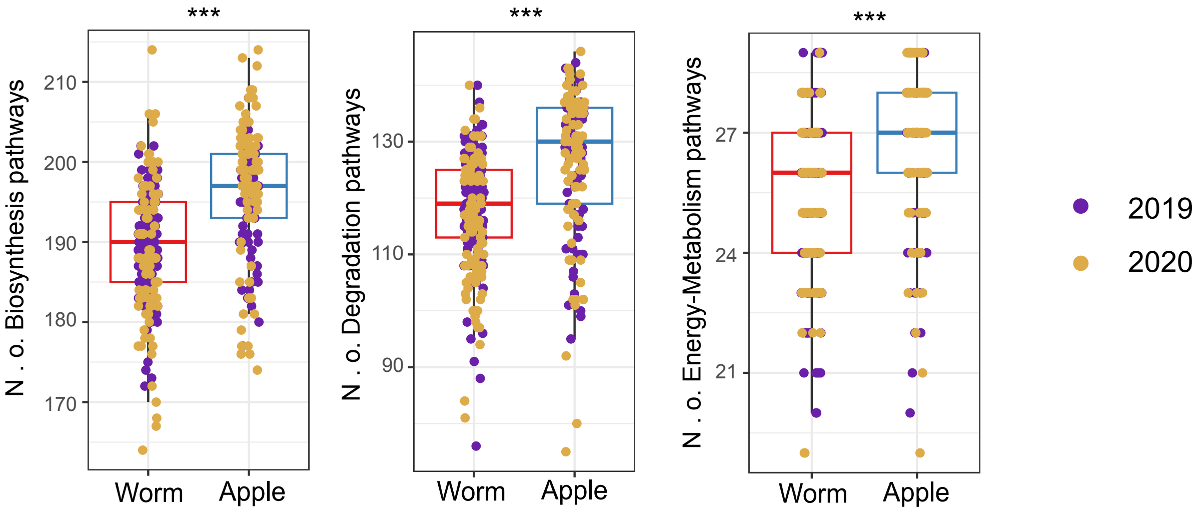

Figure S7: Significantly different, inferred pathway sub-systems between *Caenorhabditis* worm and apple microbiomes. Pathway richness analysis was inferred by PICRUSt2. Colors indicate sampling year. Statistics were performed using a linear mixed-effects model and the formula Number of pathways ~ Environment (i.e., worm or apple) + pH with the specif-ic apple for each sampling time point as random factor. P-values were adjusted using BH. Further details on the statistics are given in Table S16.

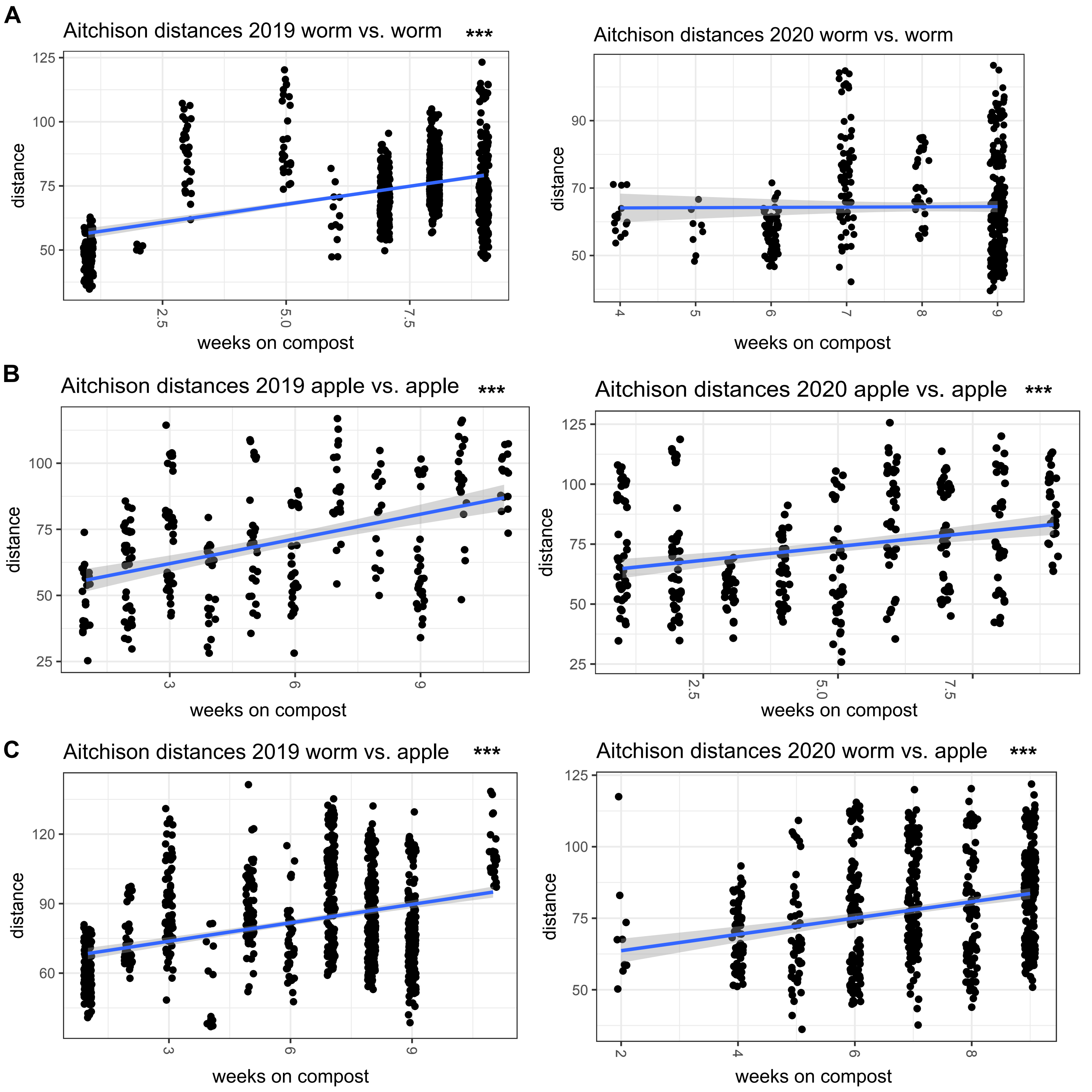

Figure S8: Relationship between weeks on compost and differentiation across microbiomes. Comparison of Aitchison distances between (A) *Caenorhabditis* worm microbiomes from the same sampling time point, but not the same apple, (B) apple microbiomes from the same sampling time point, and (C) worm and non-source apple microbiomes from the same sampling time point. Statistics were performed using a linear mixed-effects model and the formula Aitchison distance ~ weeks on compost. Further details on the statistics are given in Table S18. Since there were no or only one worm sample with successful 16S amplicon sequencing results for sampling time point 4, 10, and 11 in 2019 and sampling time points 1 - 3 in 2020, we omitted the respective data in the figure. Importantly, if data from one worm sample were available, it was not possibly to compute worm-worm dis-tances as in A, but it was possible to compute apple-worm distances in C.

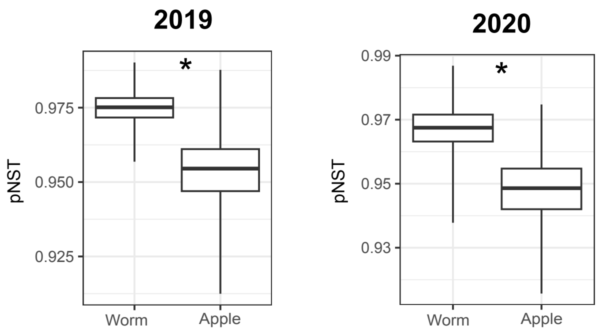

Figure S9: Analysis of the factors shaping assembly of microbiomes of *Caenorhabditis* worms and apples. pNST values for worm and apple microbiomes in 2019 and 2020. Values higher than 0.5 indicate mostly stochastic processes. Values below 0.5 indicate that assem-bly is mostly determined via deterministic processes. Asteriks are based on the p_count generated with the nst_boot() function implemented in the NST package and details are given in Table S23.

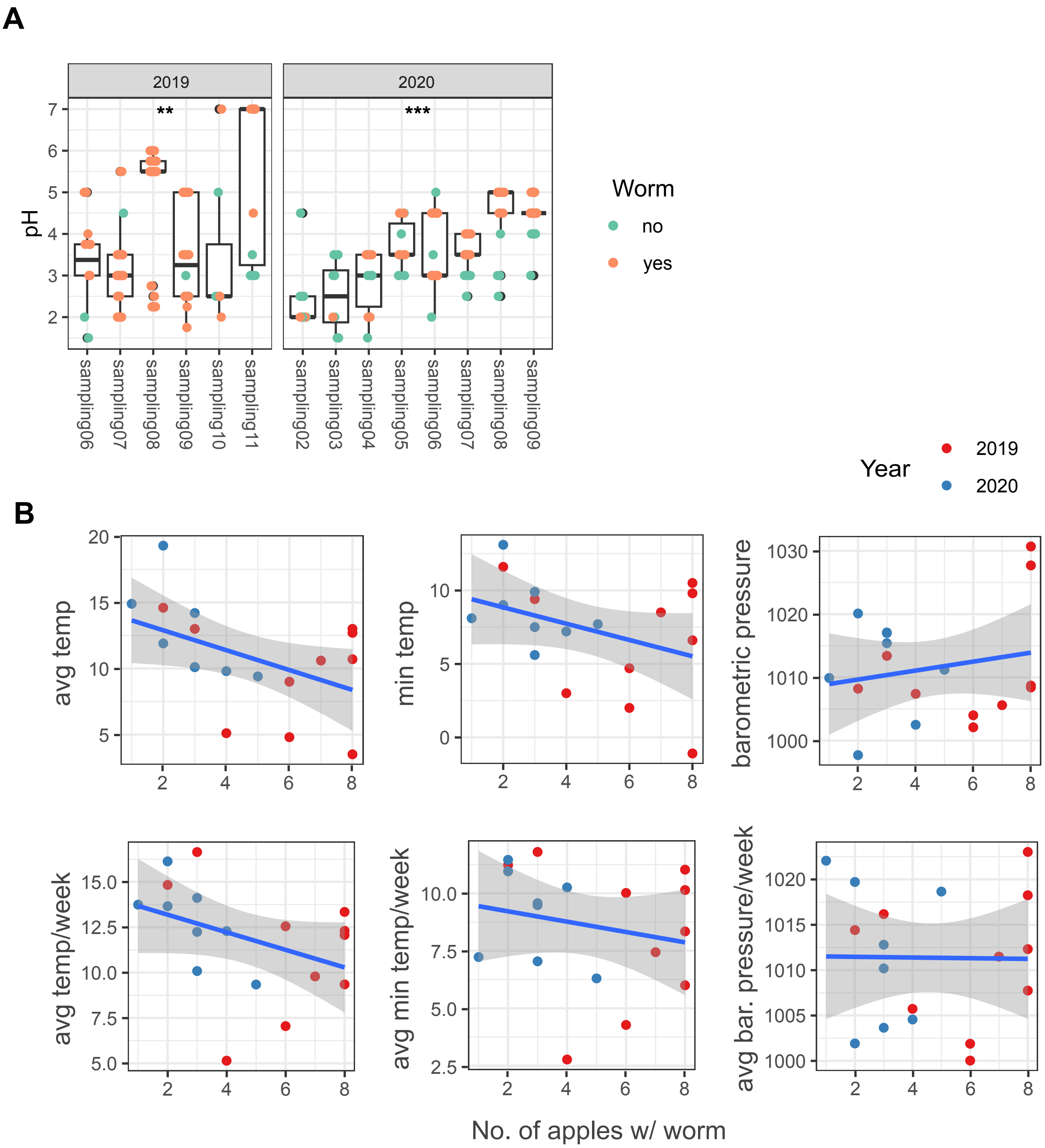

Figure S10: Detailed analysis of differences in abiotic factors between apples with and without *Caenorhabditis* worms. A: pH values of apples over sampling time (i.e., weeks ap-ples spend on compost heap). Colors indicate the presence (orange) and absence (green) of worms on apples. Asteriks indicate a significant relationship between pH and the weeks apples had spend on compost, assessed using a linear mixed model using worm presence as random varible (Table S29). B: Correlation of non-significant abiotic factors and the number of apples with worms. Colors indicate the year of the samples. The blue lines indi-cate the best fit lines for a linear relationship between both variables, while the shaded areas indicate the 95% confidence intervals for the predicted values. Statistics were per-formed using the lmer function of the lm4 package and the respective formulars are given in Table S28.

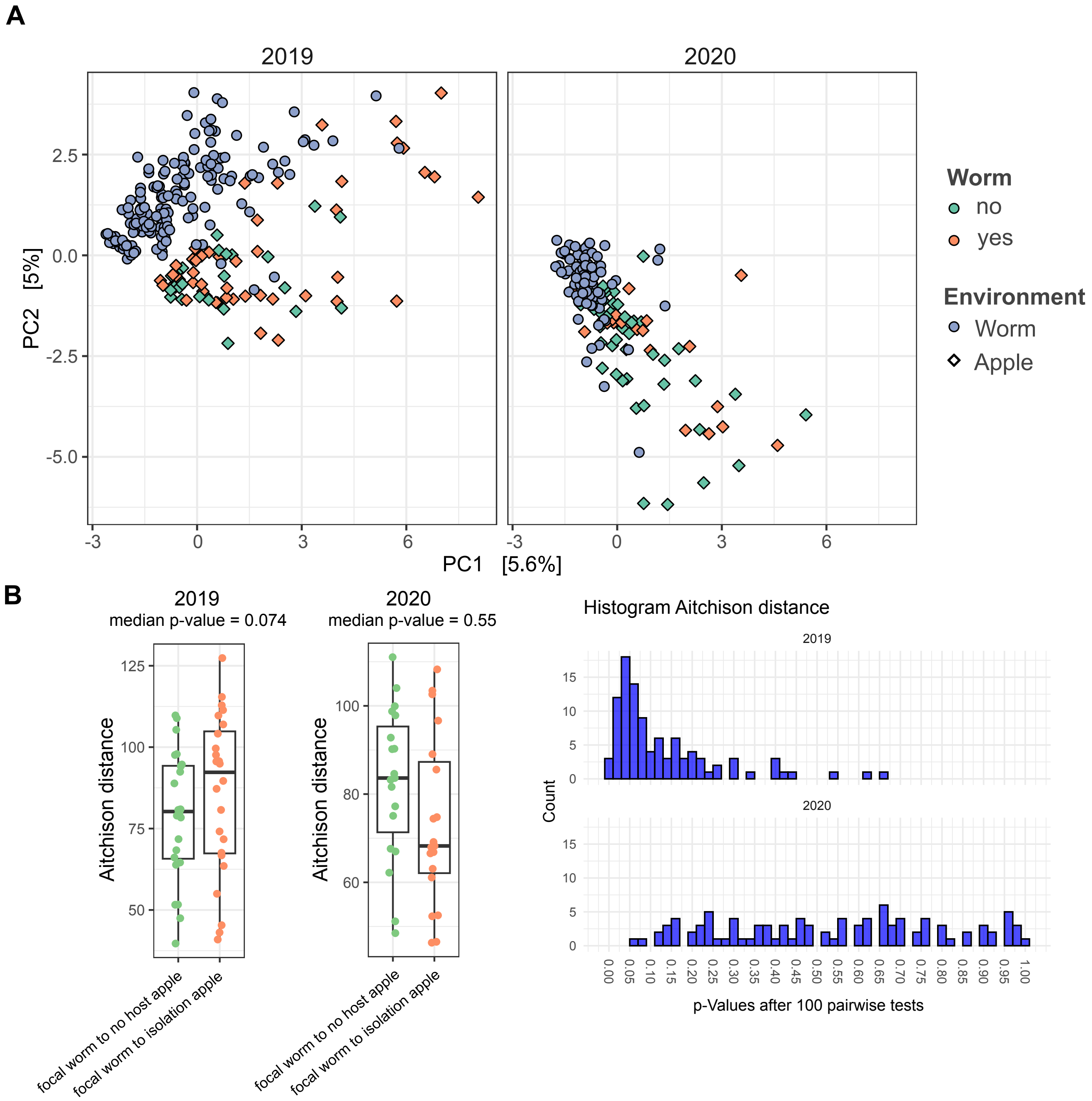

Figure S11: Relationship of microbiomes of *Caenorhabditis* worms and apples with or with-out worms. A: Beta-diversity calculated as Aitchison distance between any two microbi-omes. Shapes indicate *Caenorhabditis* worm (circles) and apple (diamonds) microbiomes. Colors indicate apples with (orange) or without worms (green) and worm microbiomes (blue). Statistics were performed using the adonis2 function of the vegan package and de-tails are given in Table S35. B: Aitchison distances between microbiomes of focal worms and “no host” apples from the same sampling time point (green) or between focal worms and their respective source apple (orange). For each focal worm, we selected a single ran-dom comparison, either with a “no host” apple or the source apple. To capture the variabil-ity of all possible pairings, we repeated this random selection process 100 times and calcu-lated a p-value for each iteration using a paired Wilcoxon test. Each figure panel presents one possible outcome with the median p-value from these repeated tests on top of each panel. The distribution of all p-values is shown as a histogram.
